# Novel digital methods for gathering intensive time series data in mental health research: scoping review of a rapidly evolving field

**DOI:** 10.1017/S0033291722003336

**Published:** 2023-01

**Authors:** Anita Schick, Christian Rauschenberg, Leonie Ader, Maud Daemen, Lena M. Wieland, Isabell Paetzold, Mary Rose Postma, Julia C. C. Schulte-Strathaus, Ulrich Reininghaus

**Affiliations:** 1 Department of Public Mental Health, Medical Faculty Mannheim, Central Institute of Mental Health, Heidelberg University, Heidelberg, Germany; 2 Department of Psychiatry and Neuropsychology, School for Mental Health and Neuroscience, Maastricht University, Maastricht, The Netherlands; 3 Centre for Epidemiology and Public Health, Health Service and Population Research Department, Institute of Psychiatry, Psychology & Neuroscience, King's College London, London, UK; 4 ESRC Centre for Society and Mental Health, King's College London, London, UK

**Keywords:** Ambulatory assessment, big data, digital phenotyping, ecological momentary assessment, experience sampling method, mental health, mobile sensing, psychopathology, sensor

## Abstract

Recent technological advances enable the collection of intensive longitudinal data. This scoping review aimed to provide an overview of methods for collecting intensive time series data in mental health research as well as basic principles, current applications, target constructs, and statistical methods for this type of data.

In January 2021, the database MEDLINE was searched. Original articles were identified that (1) used active or passive data collection methods to gather intensive longitudinal data in daily life, (2) had a minimum sample size of *N* ⩾ 100 participants, and (3) included individuals with subclinical or clinical mental health problems.

In total, 3799 original articles were identified, of which 174 met inclusion criteria. The most widely used methods were diary techniques (e.g. Experience Sampling Methodology), various types of sensors (e.g. accelerometer), and app usage data. Target constructs included affect, various symptom domains, cognitive processes, sleep, dysfunctional behaviour, physical activity, and social media use. There was strong evidence on feasibility of, and high compliance with, active and passive data collection methods in diverse clinical settings and groups. Study designs, sampling schedules, and measures varied considerably across studies, limiting the generalisability of findings.

Gathering intensive longitudinal data has significant potential to advance mental health research. However, more methodological research is required to establish and meet critical quality standards in this rapidly evolving field. Advanced approaches such as digital phenotyping, ecological momentary interventions, and machine-learning methods will be required to efficiently use intensive longitudinal data and deliver personalised digital interventions and services for improving public mental health.

## Introduction

Smartphones, sensors, and wearables may play an important role in advancing mental health research by actively or passively collecting fine-grained, multi-modal intensive longitudinal data. Active data acquisition methods include modern diary techniques, such as Experience Sampling Methodology (ESM; Csikszentmihalyi & Larson, 1987; Myin-Germeys et al., 2018) or synonymously Ecological Momentary Assessment (Shiffman, Stone, & Hufford, 2008). These methods are built on the premise that subjective experience and behaviour is situated in context and, hence, are geared towards capturing moment-to-moment variation in thoughts, feelings, and behaviours in relation to the real-world context in which they occur, i.e., in daily life, outside the research laboratory (Myin-Germeys et al., 2018), thereby, generating time-intensive longitudinal data with limited recall bias and high ecological validity. Continuous time-intensive data can also be collected passively by using dedicated, high-grade, and research-driven sensors providing objective measures of physical or physiological parameters in daily life. Passive intensive longitudinal data can be further acquired through built-in sensors of mobile devices such as smartphones and wearables (Boonstra et al., 2018). Smartphones allow for logging device usage data, application usage, and communication. These passive data collection methods come with reduced burden as they do not require active user input and allow for a high sampling frequency, enabling the detection of temporal variation in trajectories of target constructs on micro-timescales, which has been posited to provide the basis for identifying ‘digital phenotypes’ (Insel, 2017, 2018; Jain, Powers, Hawkins, & Brownstein, 2015) that may be relevant to mental ill-health (Jain et al., 2015).

Intensive longitudinal data can also be used to investigate important risk and protective factors, including candidate momentary mechanisms that may contribute to the development of mental disorders (Rauschenberg et al., 2017, 2016b; Reininghaus, Depp, & Myin-Germeys, 2016a). Allowing for the analysis of temporal variation within and between individuals, intensive longitudinal data provide detailed insights into trajectories of experience and behaviour as they occur in daily life, including their interaction with contextual or socio-environmental factors. Thus, this type of data can further our understanding of and generate evidence on, the social environment and how it contributes to our mental health (Myin-Germeys et al., 2009, 2018; Reininghaus, 2018).

Methods for collecting intensive time series data have a wide range of applications in mental health research, including digital monitoring, reporting, and feedback (Kramer et al., 2014; Rauschenberg et al., 2021a). The aim of the present scoping review is to provide an extensive overview of methods for collecting intensive longitudinal data in mental health research, including basic principles, current applications, target constructs, and statistical methods for this type of data.

## Methods

**Fig. 1.**
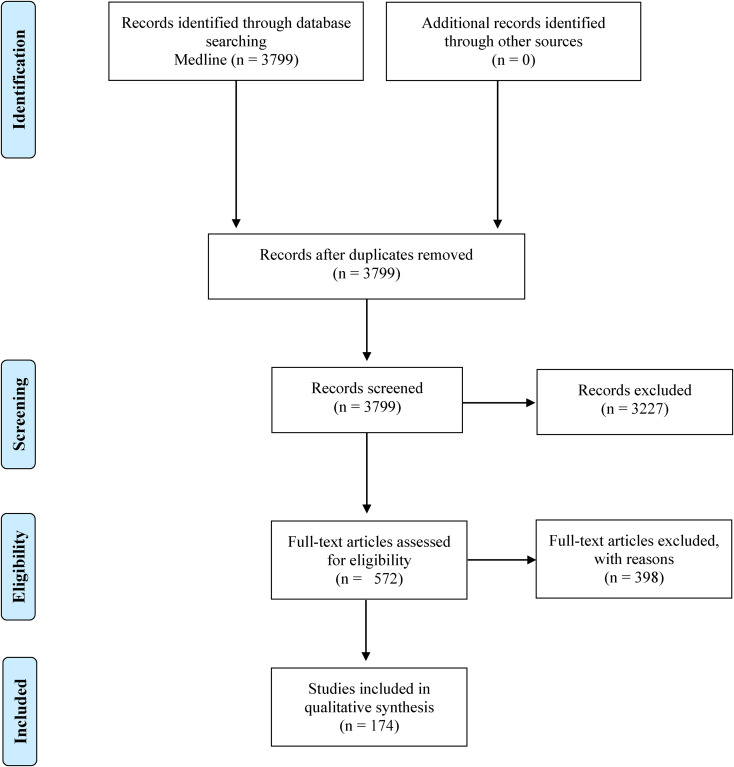
Study selection. Notes: MEDLINE was searched on 30.01.2021. Reasons for exclusion were not meeting inclusion criteria (e.g. studies investigating samples with neurological disorders). Fifteen studies were excluded as they did not meet the criteria for assessment frequency (i.e. less than 20 data points).

In January 2021, a combined search was conducted in the MEDLINE database for terms related to (a) mental disorders and, more generally, psychopathological domains (e.g. anxiety, depression), and (b) assessment methods that allow for intensive time series data collection (e.g. ESM, sensor-based technologies) (see online Supplementary Material, Table S1 for the full list of search terms). Search strings were developed and tested using MeSH terms, Boolean operators, and text words to conduct a broad search and identify relevant articles. In sum, 3799 titles and abstracts were screened for inclusion by independent reviewers (AS, CR, JSS, MD, MRP, IP, LA, CA, NM) using EndNote (TE, 2013). The references were screened and categorised as ‘eligible’, ‘query’, and ‘not eligible’. Full texts of articles categorised as eligible or query were obtained, read, and assessed against the full list of inclusion criteria. Grey literature and manuscripts from preprint servers were excluded. The study selection process is displayed as a PRISMA flow diagram in Fig. 1.

### Selection criteria

#### Inclusion criteria

Studies were included if they met the following inclusion criteria: (1) published in a peer-reviewed journal; (2) written in English, Dutch, or German; (3) contained original findings examining active (i.e. diary) or passive (i.e. sensors, mobile sensing) methods for collecting intensive time series data in daily life (i.e. defined as ⩾20 assessments per person, with a maximum time interval of one week between two assessments); (4) individuals with a diagnosis of, or at-risk for, mental disorder (i.e. first degree relatives of service users with a mental disorder, individuals with psychometric risk or an at-risk mental state); (5) published between January 2007 and January 2021; and (6) included a sample of at least 100 participants.

#### Exclusion criteria

We excluded studies that (1) used qualitative methods, single case studies, and studies with less than 100 participants, reviews, non-peer-reviewed articles, manuscripts, dissertations, PhD theses, conference proceedings, and book chapters; (2) investigated individuals from the general population without any documented psychometric risk or mental health problem; (3) focused on health-related problems without meeting criteria for a full clinical diagnosis of mental disorder; (4) investigated mHealth interventions for mental health promotion or universal prevention; (5) exclusively focused on service users that suffer neurological disorders or other medical conditions.

## Results

The search strategy yielded 3799 potential articles of interest. Following title and abstract screening, 572 full text articles were assessed for eligibility (see Fig. 1). Five studies from low- and middle-income countries were identified. They included less than 100 participants and, hence, are reported in online Supplementary Table S5. In total, 174 articles were included in the final qualitative synthesis.

### Data extraction

In total, *active data* collection methods were used in more than half of the included studies (61%, see online Supplementary Table S2). Twenty-nine publications (17%) reported findings from *dedicated sensors* (see online Supplementary Table S3), and 8 studies (5%) from *mobile sensing* (see online Supplementary Table S4). In 30 studies (17%), a *combination of active and passive methods* for collecting intensive time series data was used (see online Supplementary Table S5).

### Active data collection methods

The most commonly used active data collection method was the ESM (in 96 of 108 studies). Various sampling techniques were used in these studies, including event-contingent designs (e.g. Tasca et al., 2009), time-contingent designs (e.g. Collip et al., 2014) with (semi-) random or fixed sampling schedules, or hybrid designs (i.e. combining event- and time-contingent designs) (e.g. Smyth et al., 2009). In ESM studies, the sampling frequency ranged from three to ten assessments per day, whereas in the twelve telephone/ email studies included, the sampling frequency was between four times per day to once per week. The assessment period ranged between two days to two years (see online Supplementary Table S2). Notably, there was considerable heterogeneity in sampling designs and ESM measures.

### Applications and target constructs

Next, we extracted the most common target constructs in the identified studies.

Most studies (i.e. 75 studies), used the ESM to capture self-reported positive and negative affect (e.g. Collip *et al*. 2011a; Fitzsimmons-Craft *et al*. 2015; Hartmann *et al*. 2015; Haynos *et al*. 2015; Lavender *et al*. 2016). To this end, e.g. items from the Positive and Negative Affect Schedule (PANAS; Watson, Clark, and Tellegen, 1988) have been used. In addition, systematic variation in affective states over time (sometimes referred to as emotional instability) was frequently investigated (e.g. Johns *et al*. 2019, Solhan, Trull, Jahng, & Wood, 2009; Wonderlich *et al*. 2015). As an alternative to assessing discrete emotions, ratings of valence and arousal have been used to capture affective states (Becker, Fischer, Crosby, Engel, & Wonderlich, 2018). Affective processes have been examined in at-risk samples, or samples of service users diagnosed with various mental disorders, such as depressive (Hartmann et al., 2015; Kordy et al., 2016; Simons et al., 2015), bipolar (Tsanas et al., 2016), psychotic (Collip et al., 2011c; Lataster et al., 2011; Oorschot et al., 2012), eating (Berner et al., 2017; Fitzsimmons-Craft et al., 2015; Haynos et al., 2015), anxiety (Silk et al., 2018), and personality (Andrewes, Hulbert, Cotton, Betts, & Chanen, 2017; Chapman, Rosenthal, Dixon-Gordon, Turner, & Kuppens, 2017) disorders (see supplementary tables).

ESM has also been applied to assess cognitive processes in daily life. In psychosis research, psychotic experiences [e.g. subclinical expressions of disordered thinking, paranoia, delusions (Collip et al., 2011a; Collip et al., 2011c; Klippel et al., 2017; Reininghaus et al., 2016b; Reininghaus et al., 2016c)], threat anticipation (Klippel et al., 2017; Perez Arribas, Goodwin, Geddes, Lyons, & Saunders, 2018), and aberrant salience (Klippel et al., 2017; Reininghaus et al., 2016c) are important target constructs that have been captured using ESM. In eating disorder research, momentary assessments of social comparison processes and thoughts of compensatory behaviour added evidence to existing theories with high ecological validity (Leahey, Crowther, & Ciesla, 2011). In addition, ESM has been used to capture worrying or rumination (Khazanov, Ruscio, & Swendsen, 2019; Ruscio et al., 2015), and dissociation (Mason et al., 2017). More recently, experimental experience sampling tasks have been developed to measure momentary cognition (Reininghaus et al., 2019). However, compared to affective processes, overall, cognitive processes have been less frequently studied using ESM in mental health research. This might be due to more frequent fluctuations in cognitive constructs and to difficulty accessing these processes using traditional ESM measures (Daniels et al., 2020).

In 31 studies, ESM has also been used to assess the occurrence of specific – often dysfunctional – behavioural patterns in daily life [e.g. self-harm (Muehlenkamp et al., 2009)]. Momentary behaviour has either been assessed by event-contingent or time-contingent sampling schemes. Offering an appealing alternative to retrospective self-report measures, some studies assessed momentary behaviour, such as substance use (Fatseas, Serre, Swendsen, & Auriacombe, 2018; Serre, Fatseas, Denis, Swendsen, & Auriacombe, 2018), intoxication (Mackesy-Amiti & Donenberg, 2020; Pisetsky et al., 2016), and mode of consumption (Mackesy-Amiti & Donenberg, 2020) in the daily lives of individuals with substance use disorders. Moreover, ESM has gained increasing attention in research focusing on dysfunctional behaviour in the spectrum of eating disorders to assess, for example, restrictive eating, binge eating, and purging (Fitzsimmons-Craft et al., 2015; Lavender et al., 2016; Schaefer et al., 2020; Smyth et al., 2009; Zunker et al., 2011).

In the included studies, a strong emphasis was placed on contextual factors such as participants' current location (Mackesy-Amiti & Donenberg, 2020; Rintala, Wampers, Myin-Germeys, & Viechtbauer, 2019) and activities (Leendertse et al., 2018; Oorschot et al., 2012), but also social context [e.g. being alone or in company, interpersonal stressful events (Collip et al., 2011b; Morgan et al., 2017; Tasca et al., 2008)]. Moving beyond the assessment of context, appraisals of the context have gained increasing attention. For example, appraisals of the unpleasantness of events, activities, and social situations have been used to operationalise different types of stress (Collip et al., 2011a; Klippel et al., 2018; Peerbooms et al., 2012; Reininghaus et al., 2016b). In addition, specific processes such as social satisfaction (Collip et al., 2014) or perceived rejection (Scott et al., 2017) have been examined using ESM.

Thus, overall, ESM research to date has commonly examined a combination of affective, cognitive, and behavioural processes taking into account contextual factors to further elucidate the role of candidate momentary mechanisms (as situated in context) in the development and maintenance of mental health problems and their subjective experience (Erwin, Dennis, Coughlin, Calhoun, & Beckham, 2019; Khazanov et al., 2019; Oorschot et al., 2012). For example, the construct of affective and psychotic stress reactivity, defined as an increased intensity of negative affect and psychotic experiences in response to minor daily stressors, has been widely studied as an important putative momentary mechanism in psychosis research (Collip et al., 2011c; Frissen et al., 2014; Reininghaus et al., 2016c) and in other fields [e.g. eating disorders (Pearson et al., 2017), personality disorders (Glaser, Van Os, Mengelers, & Myin-Germeys, 2008)], and with respect to transdiagnostic phenotypes (Rauschenberg et al., 2017)). Investigating the interplay of affective processes and contexts in daily life offers the advantage of operationalising important symptom domains and their subjective experience, as has been done, for example, for negative symptoms such as anhedonia (Oorschot et al. (2012). Insights on the momentary dynamics of affective experiences and dysfunctional behaviour have advanced our understanding of the emergence of disordered eating behaviour such as restrictive or binge eating episodes and purging (Berg et al., 2017; Engel et al., 2013; Fitzsimmons-Craft et al., 2015; Goldschmidt et al., 2014; Haynos et al., 2015; Schaefer et al., 2020). ESM measures of temporal dynamics in affect, craving, and substance use behaviour in daily life can now be used to inform treatment and relapse prevention in substance use disorders (Fatseas et al., 2018; Serre et al., 2018). An overview on psychometric quality of ESM measures is provided in the supplementary material.

### Passive, sensor-based data collection methods

Overall, accelerometers were the most frequently used dedicated sensors to capture time intensive data in the included studies (i.e. in 27 of 29 studies, see online Supplementary Table S3). Only two studies reported pedometer data, i.e., estimating individuals' physical activity based on step count. Other studies using sensors capable of detecting other psychophysiological parameters, such as heart rate or electrodermal activity were not included. The assessment period in the included studies utilising dedicated sensors ranged from two hours to 22 days, and the sampling frequency was between two to 60 s epochs (see online Supplementary Table S3).

### Applications and target constructs

Most studies (i.e. 18 out of 29, see online Supplementary Table S3) used sensors to passively monitor physical activity. In the included studies, daytime physical activity was operationalised as a gradient of intensity that includes sleep, sedentary behaviour, light physical activity, moderate to vigorous physical activity, and high intensity physical activity. In addition, the included studies reported frequency and duration of specific types of physical activity, such as sedentary behaviour assessed by a pedometer (Piette et al., 2011) or accelerometer (Stubbs, Ku, Chung, & Chen, 2017), as well as gross motor activity (Difrancesco et al., 2019). Further, some of the included studies quantified physical activity on a daily basis using step count (Baerg et al., 2011), or by calculating a mean activity score from accelerometer data (Benard et al., 2019). Others (Geoffroy et al., 2019) reported the average activity during the most active 10-h period.

Another construct that has been extensively investigated using sensors is sleep (11 out of 29 studies, see online Supplementary Table S3). There are different operationalisations for sleep duration. As shown in online Supplementary Table S3, different parameters have been applied to measure sleep quality and duration including e.g., sleep efficiency and start and end time of the rest period (e.g. Blake *et al*. 2017, 2018; Fang *et al*. 2016; Goodlin-Jones, Waters, & Anders, 2009; McCrae *et al*. 2019; Owens *et al*. 2009; Robillard *et al*. 2014; Verkooijen *et al*. 2017; Wallace *et al*. 2017; Wallen, Park, Krumlauf, & Brooks, 2019; Wichniak *et al*. 2011). Among the included studies, several measures intended to assess sleep disruption, including the fragmentation index, which quantifies sleep continuity (Benard et al., 2019; Geoffroy et al., 2019). Further, two parameters were used to describe the transition period between sleep and wakefulness: sleep onset latency, which is the time required to fall asleep after going to bed (e.g. Bergwerff, Luman, & Oosterlaan, 2016; Blake *et al*. 2018), and sleep inertia, which is the time spent awake between sleep offset and getting out of bed [e.g. (Verkooijen et al., 2017)]. As demonstrated by the included studies, sensor data can also be informative to determine individuals' day-night rhythm or circadian patterns, when physical activity and sleep data are combined. The inter-daily variability (e.g. Benard *et al*. 2019; Geoffroy *et al*. 2019; Shou *et al*. 2017 is one measure to quantify consistency in sleep-wake pattern across days, whereas intra-daily variability (e.g. Geoffroy *et al*., 2019) represents an indicator for rhythm fragmentation, which relates to daytime napping or night time activity. Difrancesco et al. (2019) developed an index for circadian rhythm, also known as chronotype, or the proclivity to be asleep at a particular time of the day.

#### Mobile sensing

Only three included studies applied mobile sensing (see online Supplementary Table S4) and used log data (i.e. ingoing/outgoing calls and text messages), mobility measures (GPS, cell tower IDs, e.g. Friedmann et al., 2020) to investigate mental health outcomes. For example, Pratap et al. (2019) made use of machine learning to predict prospective group and person-level daily mood via passive smartphone data. Using GPS to capture mobility was reported as one of the most encouraging and important features in the study sample.

Five included studies investigated smartphone usage data and mainly focused on linguistic characteristics of social media usage (Birnbaum, Ernala, Rizvi, De Choudhury, & Kane, 2017; Cheng, Li, Kwok, Zhu, & Yip, 2017; Hswen, Naslund, Brownstein, & Hawkins, 2018; Hswen et al., 2017; Reece et al., 2017). The investigation of communication patterns on popular social media outlets has been used for (1) predicting the emergence of poor mental health (Eichstaedt et al., 2018; Pratap et al., 2019; Reece et al., 2017), (2) supporting early detection and intervention (Cheng et al., 2017; Hswen et al., 2018), (3) identifying individuals at-risk for, or with a diagnosis of, mental disorders (Birnbaum et al., 2017; Hswen et al., 2017), and (4) to identify important social-environmental risk and resilience factors (Birnbaum et al., 2017; Friedmann et al., 2020; Hswen et al., 2017).

### Active and passive data acquisition methods combined

The findings of our review further indicated that, to date, it is primarily sleep research that has pioneered the joint use of active and passive data acquisition methods in mental health research (i.e. 26 out of 30 included studies, see online Supplementary Table S5). The validation of measures can be accomplished by combining sensor data with self-report data e.g. on sleep (e.g. Lovato, Lack, Wright, & Kennaway, 2014; McMakin *et al*., 2019 or on other constructs such as pain (McCrae *et al*., 2019), affect (Merikangas *et al*., 2019; Wallace *et al*., 2017), or stress (Wallace et al., 2017).

### Analysis

Intensive longitudinal data typically has a multilevel structure, with repeated measurements nested within individuals. Therefore, associations among the constructs of interest can be examined on at least two levels. Analyses at the cluster level (i.e. individuals or groups) reveal information on between-person differences in individuals' average responses (e.g. those who experience more stress in their daily life are, on average, more likely to report psychotic experiences (e.g. Glaser, Van Os, Thewissen, & Myin-Germeys, 2010; Reininghaus *et al*., 2016b, 2016c). Analyses at the within-person level account for potential variability in individuals' experience and behaviour over time (i.e. from one measurement occasion to another). These analyses therefore allow for investigating temporal trajectories and uncovering event-related or context-dependent relations among the constructs under scrutiny (e.g. whether an individual has a high risk for binge eating when experiencing high levels of negative affect (e.g. Berg *et al*., 2017; Crosby *et al*., 2009; Selby *et al*., 2012).

To date, ESM in the field of mental health research has primarily reported findings based on the analyses of between-person differences i.e., aggregating ratings on target constructs across measurement occasions (e.g. Blum *et al*., 2015; Engel *et al*., 2013, Kimhy *et al*., 2014; Kuepper *et al*., 2013; Muehlenkamp *et al*., 2009; Pearson *et al*., 2016; Pisetsky *et al*., 2016). Most of the included studies conducted these types of analyses to examine the effectiveness of an intervention (e.g. comparing treatment *v.* control conditions (e.g. Chapman *et al*., 2017; Kordy *et al*., 2016; Schlam, Baker, Smith, Cook, & Piper, 2020; Silk *et al*., 2018; Simons *et al*., 2015), or to examine differences in target constructs (e.g. the experience of stress, or negative affect) across service users and healthy controls (e.g. Blum *et al*., 2015; Goldschmidt *et al*., 2013; Johns *et al*., 2019; Leraas *et al*., 2018; Morgan *et al*., 2017; Oorschot *et al*., 2012; Reininghaus *et al*., 2016b; Tsanas *et al*., 2016). However, the full benefit of analysing intensive longitudinal data collected using ESM, arguably, comes into play when also considering temporal fluctuations in the relationship between an independent variable [e.g. affective experience (Anestis et al., 2010; Berner et al., 2017; Karr et al., 2013)] and some outcome of interest [e.g. maladaptive behaviour (Anestis et al., 2010; Berner et al., 2017; Karr et al., 2013; Ruscio et al., 2015)] that unfold at the within-person level. This approach also provides a means of identifying processes and situations that precede a critical event [e.g. incidents of self-injury (Muehlenkamp et al., 2009), dietary restrictions (Engel et al., 2013), aggressive urges or behaviour (Scott et al., 2017)]. Multi-level mixed-effect models further allow for the inclusion of random effects to account for person- and day-level differences, for example, in the association between negative affect and aggressive urges by modelling random intercepts and slopes. In this way, it can be shown that there are between-person differences in complex within-person associations. For example, it has been reported (Scott et al., 2017) that an increase in perceived rejection was associated with an increase in the experience of negative affect (i.e. within-person association). This association was stronger for individuals with more pronounced borderline personality symptoms (i.e. between-person difference). Finally, examining time-lagged associations between independent variables and outcomes provide insights into the development of these associations over time. Despite this advantage, only a minority of the included studies used time-lagged analyses (24 studies, e.g. Jahng *et al*., 2011; Klippel *et al*., 2021; Wigman *et al*., 2015). Gerritsen et al. (2019), for instance, showed that high levels of activity-related stress experienced at time *t*_n−1_ predicted increases in anhedonia at time *t*_n_. Another study revealed that post-traumatic stress disorder symptom severity at time *t*_n_ was not predicted by the experience of negative affect at time *t*_n−1_, but conversely, that symptom severity at time *t*_n−1_ predicted the experience of negative affect at time *t*_n_ (Erwin et al., 2019). More recently, Klippel et al. (2021) applied cross-lagged moderated multilevel mediation analyses in order to systematically test the temporal association between momentary stress, negative affect, and psychotic experiences.

Common approaches to analyse sensor data also include multi-level modelling. However, in most studies, parameters are aggregated prior to analysis by, for example, calculating the mean score for approximating individuals' physical activity from step counts collected on several consecutive days (Benard et al., 2019). There is also a recent move towards utilising more complex methodological approaches, including supervised machine learning algorithms (e.g. Wallen *et al*., 2019, Zebin, Peek, & Casson, 2019). In particular, in long time series derived from multiple sources (e.g. several sensors) machine learning approaches using prediction models including Bayesian networks and recurrent neural networks may be applied (Koppe, Guloksuz, Reininghaus, & Durstewitz, 2019) These novel approaches are also increasingly being used for classifying individuals, e.g., into individuals with mental health problems and controls, based on mobile sensing data (Birnbaum et al., 2017). To this end, different algorithms have been applied including Support Vector Machines, Bayesian classifiers, random forest, and other decision trees.

## Discussion

The aim of this scoping review was to provide a comprehensive overview of methods used for gathering time series data in mental health research. We identified a broad range of methods, comprising self-report and various passive, sensor-based technologies. These methods have been utilised in diverse populations and settings across the full spectrum of mental ill-health. Compliance with, active and passive data collection methods in diverse clinical settings and groups was high. Most frequently studied target constructs included positive and negative affect, symptom domains, cognitive processes, sleep, and dysfunctional behaviour, as well as physical activity and social media use. Overall, our findings indicate that the included studies were highly heterogeneous in terms of design, sampling schemes, and operationalisation of target constructs – even when largely comparable constructs (e.g. negative affect) were studied. Furthermore, our review highlights that, so far, the full potential of the data captured by these methods has not been fully exploited, as often only aggregated data were analysed. The reported relationships were largely correlational in nature and only a small number of studies used more advanced statistical methods to investigate, for instance, temporality or other criteria for establishing causality. In addition, only a minority of studies applied a combination of methods.

### Methodological considerations

The current review and its findings must be viewed in light of some limitations. First, the overarching aim of the review was to provide a comprehensive overview of the various methods currently used to collect intensive longitudinal data in mental health research. However, the definition used for intensive longitudinal data may differ from field to field. In the present work, we included only studies with more than 20 assessments per person, with a maximum time interval of one week between two assessments. Although an arbitrary cut-off, this criterion aimed to exclude studies with longitudinal designs such as longitudinal cohort designs, in which data are collected over time periods of several years, and, hence, do not reflect a design for collecting intensive longitudinal data. Further, given tens of thousands of studies published on this subject, only a restricted time period, in which studies were published, was considered (i.e. January 2007 and January 2021). We focused on those with large sample size (i.e. equal to or more than 100 individuals). Thus, important studies published before 2007 or with small sample size or studies that used cost-intensive sensors (e.g. high-grade heart-rate sensors) may have failed to identify.

Second, we did not perform hand-searching and scanning of reference lists of the included articles. Also, the results were not subjected to a second independent review. While this may have led to selection bias, it is in line with recommendations for conceptual and methodological reviews of a vast and disparate literature (Lilford et al., 2001; Morgan, Burns, Fitzpatrick, Pinfold, & Priebe, 2007; Reininghaus & Priebe, 2012).

Third, the synthesis of evidence, for example, on psychometric properties of ESM measures, was hampered by use of inappropriate psychometric methods (e.g. principal component analysis for multilevel data). This reflects a limitation of the conclusion that can be drawn about the psychometric quality of ESM measures based on our review. Overall, only a relatively small number of studies investigated some psychometric domains suggested by the COnsensus-based Standards for the selection of health Measurement Instruments (COSMIN) initiative (Mokkink et al., 2010), e.g., responsiveness, interpretability and test-retest reliability were not investigated at all.

Fourth, we identified only few original articles from low- and middle-income countries despite applying more liberal eligibility criteria with regard to sample size for studies from these countries (see online Supplementary Material Table S5). This may imply that ESM studies may be less feasible or have been conducted on a smaller scale in large parts of the world, limiting the generalisability of reported findings. This may indicate the need of technology transfer or open software facilitating its application as digital monitoring and interventions may present an opportunity for global health settings by facilitating remote access to mental health services, for example, for difficult-to-reach populations (Naslund et al., 2017; Rauschenberg et al., 2021b). Future research may benefit from the use of widely available consumer rather than dedicated research devices, and facilitated by country-specific implementation strategies. Practical steps may include engagement of multiple stakeholders in user-centred designs and transdisciplinary research, including mental health practitioners, service users, digital industry, and interdisciplinary research teams.

Finally, the constructs and methods that were reported in the included studies were heterogeneous – which may further limit the generalisability of reported findings. On the one hand, this may be a result of insufficient reporting and less of an issue in future studies when recently published reporting guidelines will hopefully be followed more closely (e.g. Trull & Ebner-Priemer, 2020). On the other hand, this may in part be imminent to a rapidly growing field of research. However, with the advent of open science practices, studies in this field may be more commonly documented in a transparent and openly accessible way, as it has been common practice in other fields (e.g. randomised controlled trials) for a long time. This in turn, may provide the basis for direct replications, which are urgently needed in this rapidly evolving and methodologically diverse field. Item repositories (Hall, Scherner, Kreidel, & Rubel, 2021; Kirtley, Lafit, Achterhof, Hiekkaranta, & Myin-Germeys, 2021) may aid in the organisation, validation, and utilisation of ESM items. In the long run, open science practices may also facilitate collaboration, which may foster the use of more comparable methods (e.g. items, sampling frequencies, devices). The research community and scientific associations should work towards defining standards and reach agreement, particularly in the rapidly growing field of mobile sensing. Additional research on measurement quality and further optimisations are required to fully exploit the advancements in methods for gathering longitudinal intensive data.

### Future outlook

To date, the evidence on clinical benefits of ESM and sensor methods remains very limited. Digital monitoring may increase individual's awareness about symptoms and their interaction with the environment. As time series data allow for investigating within-person variation, patterns of associations may be revealed and personalised feedback provided based on ESM monitoring data (Rauschenberg et al., 2021a). This, in turn, may empower service users to actively participate in clinical decision-making, which is an important feature of standard health care (National Institute for Health and Care Excellence, 2021). While there is some evidence on the efficacy of ESM-derived feedback in the treatment of depression (Kramer et al., 2014), further well-designed and adequately powered RCTs are needed to examine benefits for service users.

Furthermore, ESM and sensor data have been used to trigger digital interventions known as Ecological Momentary Interventions (EMIs; Heron & Smyth, 2010; Myin-Germeys, Birchwood, & Kwapil, 2011; Myin-Germeys, Klippel, Steinhart, & Reininghaus, 2016; Reininghaus, 2018). Thereby EMIs are adaptive, and can be personalised based on the dynamics of individuals' experience and behaviour (Heron & Smyth, 2010; Myin-Germeys et al., 2016, 2018; Reininghaus, 2018; Reininghaus et al., 2016a). This also allows for testing ecological interventionist causal models (Reininghaus et al., 2016a) by examining whether targeting candidate mechanisms in daily life result in lasting changes in mental health outcomes. Remote monitoring and digital interventions recently received increasing attention as tools for tracking and mitigating the negative impact of the COVID-19 pandemic (Rauschenberg et al., 2021b). Intensive time series data – passive data collection methods in particular – may be used to monitor system- or population-level mental health or to inform more targeted programs of mental health promotion. However, as there may be a potential of scaling-up the application of ESM and sensor methods in clinical care, technical problems and adverse device effects need to be minimised, as also reflected in regulatory requirements such as those set out by the EU Medical Device Regulation.

Another aspect that has not yet come to bear, is the combination of various types of intensive time series data that may help advance our understanding of critical determinants, developmental candidate mechanisms, and the persistence of mental health problems. The combination of ESM with sensor-based assessments may enable a deeper understanding of context specific influences. Furthermore, mobile sensing and digital phenotyping may have the potential to advance mental health research, particularly when passive data is collected concurrently with self-report data (Myin-Germeys et al., 2018; Trull & Ebner-Priemer, 2014). However, this also bears privacy risks and users need to be adequately informed and educated about the applied privacy settings. These methods may therefore empower users also with respect to data and digital health literacy when applied according to current regulations. Careful attention needs to be paid to data safety and privacy issues and users need to be adequately informed about privacy settings of sensor methods. It is notable that only very few included studies have taken advantage of the potential for combining active and passive methods for collecting intensive time series data. This is true even though it opens up new avenues for more context-sensitive sampling strategies that link experience to specific events or behavioural patterns, such as GPS-triggered ESM reports (Tost et al., 2019). However, the added value of combining active and passive data collection methods must be demonstrated in future studies.

## Conclusion

While technological advancements have significantly increased the opportunities for collecting intensive time series data in mental health research, the field continues to face critical challenges in the years to come. This includes current reporting practices, the use of insufficient statistical approaches to fully exploit the potential of multimodal longitudinal data, and establishing best practices for studies that purposefully combine various modes of data collection. Open science practices have the potential to increase transparency, generalisability, and reproducibility in this rapidly evolving field. Further, the field requires a consensus on the operationalisation of constructs and robust evidence on the psychometric quality of existing measures are critical next steps. The use of ESM and other intensive longitudinal data collections methods have enormous potential for digital monitoring and personalised feedback on service users' experience and behaviour that can be used meaningfully by service users and clinicians. This may include empowering individuals with mental health conditions to more effectively manage their mental and physical health, as well as informing and extending face-to-face sessions to real-world situations and more personalised treatment based on adaptive, ecological momentary interventions. How the research community will address these opportunities and challenges will determine whether the digital transformation of public mental health provision results in tangible benefits for users, carers, and practitioners.

## Supplementary material

For supplementary material accompanying this paper visit https://doi.org/10.1017/S0033291722003336. click here to view supplementary material
