# Supplementary material for: Novel digital methods for gathering intensive time series data in mental health research: scoping review of a rapidly evolving field
Source: Psychol Med. 2022 Nov 15;53(1):55–65. doi: 10.1017/S0033291722003336 (PMC9874995; doi:10.1017/S0033291722003336)
Supplement: Supplementary file 1 [file S0033291722003336sup001.docx]

**SUPPLEMENTARY MATERIALS**

[**Quality assessment** 2](#_Toc110499712)

[**Psychometric quality of ESM measures** 2](#_Toc110499713)

[**Psychometric quality of sensor measures** 3](#_Toc110499714)

[**Table S1. Search strategy.** 5](#_Toc110499715)

[**Table S2. Summary of included studies using the diary methods, ESM or telephone calls.** 8](#_Toc110499716)

[**Table S3. Summary of included studies using sensors.** 37](#_Toc110499717)

[**Table S4. Summary of included studies using mobile sensing.** 43](#_Toc110499718)

[**Table S5. Summary of studies from low and middle income countries (China, Brazil) with less than 100 participants.** 57](#_Toc110499719)

[**References** 59](#_Toc110499720)

# **Quality assessment**

We evaluated the quality of measures in accordance with the COnsensus-based Standards for the selection of health Measurement Instruments (COSMIN) initiative (Mokkink *et al.*, 2010). Measurement compliance, measures of reliability and validity were evaluated for ESM and sensor methods.

## **Psychometric quality of ESM measures**

*Compliance*

Overall, one quarter of the included articles (i.e., 27 studies) have not reported on compliance or measurement quality (e.g., psychometric properties) (see Table S2). While some studies defined criteria for a minimum number of completed assessments needed for participants to be included in analytic samples (Collip *et al.*, 2011a, Kuepper *et al.*, 2013, Lataster *et al.*, 2011, Leendertse *et al.*, 2018), the majority of studies reported satisfactory compliance rates with 79% mean compliance across all ESM studies. A similar compliance rate has been reported in a recent meta-analyses of ESM studies (Vachon et al., 2019), in which a smaller number of ESM items and, hence, shorter duration of each ESM assessment period was associated with better compliance, suggesting that keeping assessment burden at a manageable level may ensure good compliance. Investigating compliance in a large, transdiagnostic dataset, Rintala, Wampers, Myin-Germeys, and Viechtbauer (2019) found comparable compliance rates for different clinical samples and the general population (83%) – except for individuals with psychosis, who showed reduced compliance (70%). In addition, the authors reported similar compliance rates irrespective of the number of ESM items used (ranging from 42 to 52 items per measurement).

*Reliability*

About two thirds of the studies included in this review have reported reliability indicators, including measures of internal consistency (see Table S2). No study reported the test-retest reliability. Studies that reported on other types of reliability frequently referred to previously published data (Collip *et al.*, 2011b, Klippel *et al.*, 2017, Lataster *et al.*, 2011, Oorschot *et al.*, 2012, Peerbooms *et al.*, 2012, Reininghaus, Depp, & Myin-Germeys, 2016a, Thewissen *et al.*, 2011), or provided information on whether between-person differences were reliably captured (across individual measurements), most commonly expressed as internal consistency by reporting Cronbach's alpha (Becker, Fischer, Crosby, Engel, & Wonderlich, 2018, Berg *et al.*, 2017, Engel *et al.*, 2013, Frissen *et al.*, 2014, Glaser, Van Os, Mengelers, & Myin-Germeys, 2010, Goldschmidt *et al.*, 2014b, Hartmann *et al.*, 2015, Haynos *et al.*, 2015, Lavender *et al.*, 2013b, Leraas *et al.*, 2018, Pisetsky *et al.*, 2016, Wonderlich *et al.*, 2015). While the reliability of measurements captured using ESM should be examined separately for the within-person and the between-person level, only a small number of studies (e.g., Scott *et al.* (2017), Smyth *et al.* (2009), Sperry, Walsh, & Kwapil (2020)) provided indicators suitable to reflect within-person reliability (i.e., whether within-person measurements captured the target construct reliably over time).

*Validity*

Approximately half of the studies included in this review provided information on validity of the data collected using ESM (see Table S2).  In the included studies, criterion validity and construct validity were the most reported types of validity. The criterion validity of experience sampling measures that capture affective experiences has been assessed by identifying individual differences in affective variability patterns between specific diagnostic conditions (e.g., Leraas et al.,(2018)). Similarly, criterion validity of experience sampling measures can be determined by whether ESM measures accurately predict the occurrence of specific types of behaviour (e.g., Haynos *et al.* (2017), Tasca *et al.* (2009)). Construct validity of experience sampling measures has been established more frequently through correlation coefficients with established questionnaire measures (Ruscio *et al.*, 2015, Scott *et al.*, 2017, Solhan, Trull, Jahng, & Wood, 2009, Tasca *et al.*, 2009) or interview ratings (Klippel *et al.*, 2017). Eight included studies examined the structural validity, for example, by conducting factor analyses (e.g., Geschwind, Peeters, Drukkker, van Os, & Wichers (2011), Hartmann *et al.* (2015), Thewissen *et al.* (2011), Udachina, Bentall, Varese, & Rowse (2017)).

## **Psychometric quality of sensor measures**

*Compliance*

Passive data acquisition usually comes with high compliance rates. The mean compliance rate in studies applying sensors was 82%, however the majority of studies did not report on compliance. Furthermore, some studies applied minimum criteria for compliance to ensure data quality and reliability, e.g., a minimal wear time of 21 hours per day (Wichniak *et al.*, 2011), 16 hours per day and per night on at least one week day and one weekend day (Difrancesco *et al.*, 2019), or a minimum number of consecutive days (Bergwerff, Luman, & Oosterlaan, 2016, Pillai, Steenburg, Ciesla, Rothm & Drake, 2014, Wallace *et al.*, 2017). With respect to data quality, several studies reported relatively small amounts of missing data of 5.3% (Wallace *et al.*, 2017) and 6.1% (Blake *et al.*, 2018) or exclusion rates of 7% (Pillai *et al.*, 2014). In addition, in ambulatory sleep research, data from the first night are usually excluded from the analysis to avoid potential alterations of sleep given that participants may not be used to wearing a sensor (Pillai *et al.*, 2014).

*Reliability*

No included study reported indicators of reliability of the measures for physical activity or sleep. However, some studies referred to previous literature supporting the reliability and validity of used sensors (e.g., Bergwerff *et al.* (2016), Owens *et al.* (2009), Pillai *et al.* (2014), Sauchelli *et al.* (2015)).

*Validity*

Several studies aimed to investigate construct validity of measures derived from actigraphy. For instance, Bracht et al. (2012) reported an association between actigraphy data and the motor domain of the Bern Psychopathology Scale in individuals with psychosis, while another study (Gregory *et al.*, 2011) revealed associations between sleep items of the Child Behaviour Checklist and sleep onset latency derived from actigraphy in children with affective disorders. There was some evidence for a dose-response relationship as individuals with higher symptom levels of depression and anxiety were less active, slept more, and showed smaller differences between day-, and night-time activity, suggesting good ecological validity of actigraphy-based sleep data (Difrancesco *et al.*, 2019). Similarly, Wichniak et al. (2011) found that higher symptom levels in individuals with psychosis were related to lower activity.

# **Table S1. Search strategy.**

**MEDLINE**

Database: Ovid MEDLINE(R) <1946 to January Week 4 2021>

Search Strategy:

--------------------------------------------------------------------------------

1 depressi*.tw. (341924)

2 anxi*.tw. (182280)

3 phobi*.tw. (10370)

4 psychosis.tw. (32279)

5 psychotic.tw. (29790)

6 schizophrenia.tw. (95956)

7 paranoi*.tw. (7493)

8 hallucinat*.tw. (12461)

9 delusion*.tw. (9660)

10 bipolar*.tw. (55426)

11 mania.tw. (9122)

12 "personality disorder*".tw. (17922)

13 "Attention Deficit*".tw. (25640)

14 "Conduct Disorders".tw. (655)

15 "substance-related disorder*".tw. (477)

16 "substance disorder*".tw. (317)

17 "eating disorder*".tw. (17078)

18 "binge-eating".tw. (5019)

19 bulimi*.tw. (7750)

20 anorexi*.tw. (30080)

21 or/1-20 (670022)

22 "mobile health".tw. (2369)

23 mHealth.tw. (1958)

24 "app-based".tw. (336)

25 "mobile app*".tw. (2670)

26 "mobile-based".tw. (295)

27 "phone-based".tw. (854)

28 smartphone.tw. (6398)

29 "smartphone-based".tw. (1139)

30 smartwatch.tw. (154)

31 wearable.tw. (7506)

32 "mobile device".tw. (786)

33 "digital tool".tw. (66)

34 "mobile-sensing".tw. (43)

35 "mobile sensing".tw. (43)

36 "global positioning system".tw. (1407)

37 "position sens*".tw. (2002)

38 geotrack*.tw. (8)

39 "geo track*".tw. (2)

40 "location track*".tw. (76)

41 "step count".tw. (805)

42 pedometer.tw. (1796)

43 pulsometer.tw. (11)

44 actigraphy.tw. (3088)

45 accelerometer.tw. (8313)

46 "motion sens*".tw. (2024)

47 gyroscope*.tw. (765)

48 "use log*".tw. (344)

49 logging.tw. (2051)

50 "social media".tw. (8302)

51 facebook.tw. (2699)

52 twitter.tw. (2342)

53 instagram.tw. (374)

54 "text mining".tw. (1858)

55 "optical character recognition".tw. (92)

56 "optical character reader".tw. (4)

57 "character recognition".tw. (257)

58 "word recognition".tw. (4038)

59 "speech recognition".tw. (3282)

60 "voice recognition".tw. (444)

61 "speech to text".tw. (57)

62 sensor.tw. (67538)

63 "digital monitoring".tw. (51)

64 "mood tracking".tw. (12)

65 "digital phenotyping".tw. (53)

66 "experience sampling method".tw. (310)

67 "ecological momentary assessment".tw. (1213)

68 "ecological momentary intervention".tw. (28)

69 "naturalistic assessment".tw. (33)

70 "intensive time series".tw. (14)

71 "intensive longitudinal data".tw. (64)

72 "time series analysis".tw. (4643)

73 or/22-72 (129139)

74 21 and 73 (4250)

75 limit 74 to yr="2007 -Current" (3799)

***************************

# **Table S2. Summary of included studies using the diary methods, ESM or telephone calls.**

| **Number** | **Reference** | **Population** | **Sampling scheme** | **Sampling frequency of random prompts** | **Assessment period** | **Primary outcome is a time intensive measure** | **Data aggregated^1^** | **Measurement quality (compliance, % data)** | **Psychometric properties**  **(reliability, validity)** |
| --- | --- | --- | --- | --- | --- | --- | --- | --- | --- |
| **ESM** | | | | | | | | | |
| 1 | Andrewes *et al.* (2017) | Patients [BPD] | Semi-random | 6 | 6 days | Yes (NA, behavior) | Yes | Compliance: 52%  5% of participants were excluded due to failure to complete any reports. Twenty-two participants (21%) were given the phone for another 6 days as they filled < 20 prompts. | *Reliability:* Referred to literature for reliability of positive and negative affect scale.  *Validity:* Referred to the literature for structural characteristics of the measure. |
| 2 | Anestis *et al.* (2010) | Patients [BN] | Hybrid ( semi-random, interval-contingent, event-contingent) | 6 | 14 days | Yes (PA, NA, behavior) | Yes | Not reported. | *Reliability:* Not reported.  *Validity:* Not reported. |
| 3 | Becker *et al.* (2018) | Patients [BN] | Semi-random | 6 | 14 days | Yes (PA, NA, eating behaviour) | Yes | *Compliance:* 86% (median = 90%).  75% of the participants responded to 83% or more of the signals. | *Reliability:* Internal consistency reported for NA (Cronbach α=.92) and for anger/hostility (Cronbach α=.89).  *Validity:* Not reported. |
| 4 | Berg *et al.* (2013) | Patients [BN] | Hybrid (semi-random, event-contingent, interval-contingent) | 6 | 14 days | Yes (NA) | Yes | *Compliance:* 86% (median: 90%). | *Reliability:* Internal consistency reported for NA (Cronbach α=.92). Cronbach alphas for the abbreviated lower-order NA subscales were α=.80 (fear), α=.89 (guilt), α=.79 (hostility), and α=.81 (sadness).  *Validity* reported based on Confirmatory Factor Analysis with ESM data (PANAS negative affect scale). |
| 5 | Berg *et al.* (2017) | Patients [AN; EDNOS-AN; BN] | Hybrid (semi-random, event-contingent) | 10 | 15 | Yes (NA) | Yes | Not reported. | *Reliability:* Internal consistency was reported for NA in study 1 (Cronbach α=.94) and for NA in study 2 (Cronbach α=.92).  *Validity*: Not reported. |
| 6 | Berner *et al.* (2017) | Patients [BN] | Semi-random | 6 | 14 days | Yes (PA, NA, behavior) | Yes | Compliance: 86% | *Reliability:* Internal consistency of NA (Cronbach α=.92), and PA (Cronbach α=.91) reported.  *Validity:* Not reported. |
| 7 | Block *et al.* (2020) | Patients [MDD; social anxiety]; healthy controls | Semi-random | 6 | 7 days | Yes (stress, sleep quality) | Yes | *Compliance:* 93% | *Reliability:* Internal consistency of the openness and engagement items was r= .86.  *Validity:* Not reported. |
| 8 | Blum *et al.* (2015) | Patients [schizophrenia]; healthy controls | Random | 10 | 2 days | Yes (NA) | Yes | *Compliance*: 75% (patients); 89% (controls).  2 % of participants responded to < 30% of reports and their data was excluded. | *Reliability:* Not reported.  *Discriminant validity*: Comparison of real-time (ESM) and retrospective (questionnaire) depressed mood ratings suggested that both indices are valid measures of depressed mood.  *Convergent validity*: Both measures of depressed mood were significantly correlated with social support and quality of life suggesting convergent validity of the depressed mood measures. |
| 9 | Chapman *et al.* (2017) | Patients [BPD; MDD]; healthy controls | Semi-random | 8 | 6 days | Yes (PA, NA) | Yes | *Compliance:* At pre-instruction phase: 66%. At instruction phase: 67%. At post-instruction phase: 60%. | *Reliability:* Not reported.  *Validity:* Not reported. |
| 10 | Collip *et al.* (2011a) | High-risk population [psychotic disorder]; healthy controls | Random | 10 | 6 days | Yes (event stress, psychotic experiences, NA) | Yes | *Compliance*: 68% (high-risk), 76% (controls).  10% of participants filled < 20 reports and their data were excluded. | *Reliability:* Not reported.  *Validity:* Reports were considered valid when subjects responded within 15 min after the beep, as determined by comparing the actual beep time with the reported time of completion. |
| 11 | Collip *et al.* (2014) | Individuals at-risk from the general population [psychosis] | Random | 10 | 5 days | Yes (PA) | Yes | *Compliance:* 77%.  At least 1/3 valid responses required to be included. Number of excluded participants not mentioned. | *Reliability:* Internal consistency reported for PA (Cronbach α=.86).  *Validity:* Not reported. |
| 12 | Collip *et al.* (2011b) | Patients [psychotic disorder]; individuals at-risk from general population [psychosis] | Random | 10 | 6 days | Yes (paranoia) | Yes | *Compliance:* 73%.  16% of participants were excluded from the analyses, of these 22 participants terminated the study prematurely because of severity of psychotic symptoms (n=11), not being able to understand the instructions (n=5) or lack of cooperation (n=6).Three individuals were excluded as they filled <20 prompts. | *Reliability:* Very good reliability for the paranoia measure (Cronbach α= .82), perceived social threat measure (Cronbach α=.70).  *Validity:* Reports were assumed to be valid when participants responded to the beep within 15 min. |
| 13 | Collip *et al.* (2011c) | Patients [psychotic disorder]; healthy controls | Random | 10 | 6 days | Yes (stress reactivity) | Yes | *Compliance:* 73%.  3% of participants were excluded from the analysis as they answered < 20 prompts. | *Reliability:* Internal consistency was reported for NA (Cronbach α=.83), for psychotic symptoms (Cronbach α=.72), for hallucinations (Cronbach α=.77), and for delusions (Cronbach α=.68).  *Validity:* Not reported. |
| 14 | Crosby *et al.* (2009) | Patients [BN] | Hybrid (semi-random, event-contingent) | 6 | 14 days | Yes (NA, eating behaviour) | Yes | Not reported.  10 (out of 143) participants were excluded from analysis due to drop-out (7 participants) and non-compliance with EMA protocol (10 participants). | *Reliability:* Internal consistency was reported for NA (Cronbach α=.92).  *Validity:* Not reported. |
| 15 | De Young *et al.* (2013b) | Patients [BN; AN] | Hybrid (semi-random, event-contingent, interval-contingent) | 6 | 14 days | Yes (NA) | Yes | Not reported for semi-random prompts. | *Reliability:* High internal consistency for NA (Cronbach α=.91), and of the guilt facet (Cronbach α=.84).  *Validity:* Not reported. |
| 16 | De Young *et al.* (2013a) | Patients [AN; EDNOS-AN] | Hybrid (semi-random, event-contingent, interval-contingent) | 6 | 14 days | Yes (eating behaviour) | Yes | Not reported. | *Reliability:* Not reported.  *Validity:* Not reported. |
| 17 | De Young *et al.* (2014) | Patients [AN; EDNOS-AN] | Hybrid ( semi-random + interval-contingent, event-contingent) | 6 | 14 days | Yes (PA, NA, behavior) | No | Not reported. | *Reliability:* Not reported.  *Validity:* Not reported. |
| 18 | Engel *et al.* (2013) | Patients [AN; EDNOS-AN] | Hybrid (semi-random, event-contingent, interval-contingent) | 6 | 14 days | Yes (PA, NA) | Yes | *Compliance:* 77%. | *Reliability:* Internal consistency reported for PA (Cronbach α=.92) and NA (Cronbach α=.94).  *Validity:* Referred to literature. |
| 19 | Engel *et al.* (2007) | Patients [BN] | Hybrid (semi-random, event-contingent, interval-contingent) | 6 | 14 days | Yes (eating behaviour) | Yes | *Compliance:* 90%. | *Reliability:* Internal consistency reported for profile of mood states (Cronbach α=.89).  *Validity:* Not reported. |
| 20 | Erwin *et al.* (2019) | Patients [PTSD] | Hybrid (semi-random, event-contingent) | 6 | 7 days | Yes (NA) | No | *Compliance:* 33%  (due to mistakenly initiating entries, technical difficulties, and failure to finish entries). | *Reliability:* Internal consistency reported for NA (Cronbach α=.91) and PTSD symptoms (Cronbach α=.87).  *Validity:* Not reported. |
| 21 | Fatseas *et al.* (2018) | Patients [SUD] | Fixed | 4 | 14 days | Yes (NA, behaviour) | No | *Compliance:* 83%. | *Reliability:* Not reported.  *Validity:* Referred to the literature: EMA items have been validated in other studies with a similar same sample. |
| 22 | Fitzsimmons-Craft *et al.* (2015) | Patients [AN; EDNOS-AN] | Hybrid ( semi-random + interval contingent, event-contingent) | 6 | 14 days | Yes (PA, NA) | No | *Compliance*: 87%.  Compliance with end-of-day ratings was 89%.  2% of participants had a compliance < 50% and their data were excluded from analyses. | *Reliability:* Internal consistency was reported for PA (Cronbach α=.92), and for NA (Cronbach α=.94).  *Validity:* Not reported. |
| 23 | Frissen *et al.* (2014) | Patients [non-affective psychotic disorder]; at-risk [first degree relatives of patients]; healthy controls | Semi-random | 10 | 6 days | Yes (NA, stress) | Yes | Not reported.  Exclusion of participants due to not answering >20 prompts: 5 % of patients, 16 % of relatives, and 3% of controls. | *Reliability:* High internal consistency of negative affect scale (Cronbach α= .81). To determine the reliability of the completed reports, the times at which the watch emitted a signal and the times at which subjects completed the reports were compared.  *Validity:* Not reported. |
| 24 | George *et al.* (2018) | Individuals at risk [mental health problems] | Semi-random | 3 | 30 days | Yes (behaviour, symptoms) | No | *Compliance*: 92%. | *Reliability:* Daily depression (ICC = 0.42), anxiety (ICC = 0.37), ADHD (ICC = 0.35) and conduct problem (ICC = 0.22) symptom measures were created by summing the total number of symptoms across the day.  *Validity:* ESM items were derived from validated questionnaires. |
| 25 | Geschwind *et al.* (2011) | High-risk population [MDD] | Semi-random | 10 | 6 days | Yes (PA) | Yes | Compliance: 82%.  559 entries (4 %) were excluded because completion times fell outside the pre-determined window of 15 minutes after the beep. One participant was excluded due to completing < 20 entries. | *Reliability:* Internal consistency was reported for PA (Cronbach α=.89), and for NA (Cronbach α=.86).  *Validity*: PCA was used to generate a factor representing PA and a factor representing NA. One mood item (“I feel relaxed”) was not included in the PA factor due to low factor loadings. |
| 26 | Glaser *et al.* (2008) | Patients [BPD; psychosis spectrum disorder]; healthy controls | Semi-random | 10 | 6 days | Yes (NA) | Yes | *Compliance:* 77%.  Exclusion of participants with less than 20 reports. | *Reliability:* Internal consistency reported for activity related stress (Cronbach α=.69), NA (Cronbach α=.94), and PA (Cronbach α=.97).  *Discriminant validity:* NA and PA, were significantly, but moderately, negatively correlated. *Construct validity:* Event-related stress and activity related stress, were significantly, but low, correlated. |
| 27 | Glaser *et al.* (2010) | Patients [BPD; cluster C personality disorder; psychotic disorder]; healthy controls | Semi-random | 10 | 6 days | Yes (PA, NA, symptoms) | Yes | Not reported.  10% of patients with BPD and 10% of individuals with cluster C personality disorder were excluded because they answered < 20 prompts. | *Reliability:* Internal consistency was reported for PA (Cronbach α=.97), for NA (Cronbach α=.90) and for psychotic experiences (Cronbach α=.86).  *Validity:* Referred to the literature for evidence on validation of measures. |
| 28 | Granholm *et al.* (2013) | Patients [schizophrenia; schizoaffective disorder] | Fixed | 4 | 7 days | Yes (behaviour) | No | *Compliance:* 72%. | *Reliability:* Not reported.  *Validity:* The question content and format, as well as the sampling schedules, were validated in a subsample (n = 56). |
| 29 | Gerritsen *et al.* (2019) | Patients [psychosis]; high-risk population [psychosis] | Semi-random | 10 | 4 to10 days | Yes (stress, anhedonia) | No | *Compliance:* 70%  52 out of 167 participants did not meet the requirements for data inclusion (i.e. reports given within 15 min after a beep and at least 1/3 of the prompts had to be answered. | *Reliability:* Internal consistency was reported for activity related stress (Cronbach α=.73).    *Validity:* ESM Items were developed to maximize face validity, and have been shown to possess construct and discriminant validity. |
| 30 | Goldschmidt *et al.* (2013) | Patients [BN] | Hybrid (semi-random, event-contingent, interval-contingent) | 6 | 14 days | Yes (NA) | Yes | Not reported. | *Reliability:* High internal consistency for NA (Cronbach α=.92), and for anxiety (Cronbach α=.80).  *Validity:* Not reported. |
| 31 | Goldschmidt *et al.* (2014a) | Patients [AN] | Hybrid (semi-random, event-contingent, interval-contingent) | 6 | 14 days | Yes (eating behaviour) | Yes | *Compliance:* 87%  77% of all signals were responded to within 45 minutes.  Compliance with end-of-day ratings: 89%. | *Reliability:* Internal consistency was reported for NA (Cronbach α=.94), for guilt (Cronbach α=.86) and for fear (Cronbach α=.92).  *Validity:* Momentary PANAS items were chosen based on high factor loadings, but PCA was not reported. |
| 32 | Goldschmidt *et al.* (2014b) | Patients [BN] | Hybrid (semi-random, event-contingent, interval-contingent) | 6 | 14 days | Yes (NA, behaviour) | No | *Compliance:* 86%. | *Reliability:* Internal consistency reported for NA (Cronbach α=.92). Stressful events were categorized by two independent raters (kappa=.78).  *Validity:* Not reported. |
| 33 | Goldschmidt *et al.* (2015) | Patients [AN] | Hybrid (semi-random + interval-contingent, event-contingent) | 6 | 14 days | Yes (eating behavior, NA, stress) | Yes | Not reported. | *Reliability*: Not reported.  *Validity:* Not reported. |
| 34 | Hartmann *et al.* (2015) | Patients [MDD] | Semi-random | 10 | 5 days (Baseline),  3 days (over a course of 6 week intervention),  5 days (Post-intervention) | Yes (PA, NA) | Yes | *Compliance:* 79% (at baseline and post-intervention).  ESM periods with < 30% of beeps completed were excluded from the analyses, leading to the exclusion of one baseline ESM period, four post ESM periods, and two periods during the intervention (i.e., exclusion of 83 observations (<1%)). | *Reliability:* Internal consistency was reported for PA (Cronbach α=.74) and for NA (Cronbach α=.54).  *Validity:* PCA of ESM items was conducted: identified two factors accounting for 46% of the variance. |
| 35 | Haynos *et al.* (2015) | Patients [AN; EDNOS-AN] | Hybrid ( semi-random + interval contingent, event-contingent) | 6 | 14 days | Yes (PA, NA, mood) | No | Participants provided EMA recordings on 13 days on average. No further information on compliance reported. | *Reliability:* Internal consistency was reported for PA (Cronbach α=.92), for NA (Cronbach α=.94) and for the profile of mood states (Cronbach α=.92).  *Validity:* Not reported. |
| 36 | Haynos *et al.* (2017) | Patients [AN] | Hybrid (semi-random, event-contingent, interval-contingent) | 6 | 14 days | Yes (PA and NA, eating behaviour) | Yes | Not reported. | *Reliability:* Internal consistency was reported for joviality (Cronbach α=.91), for self-assurance (Cronbach α=.83), for guilt (Cronbach α=.93), and for fear (Cronbach α=.86).  *Validity:* Multilevel CFA conducted to evaluate factor structure of ESM PA and NA items indicating a 4 factor structure. |
| 38 | Heininga *et al.* (2017) | Individuals at-risk from general population [anhedonia]; healthy controls | Semi-random | 3 | 30 days | Yes (PA) | Yes | *Compliance:* 93%, no difference between groups. | *Reliability:* Internal consistency of PA scale (Cronbach α=.94), and inter-item correlation of the two high-arousal and low-arousal items (r=.90, r=.91) reported.  *Validity:* Not reported. |
| 39 | Jahng *et al.* (2011) | Patients [BPD; MDD] | Semi-random | 6 | 28 days | Yes (PA, NA, mood) | No | *Compliance:* 45% - 100%.  The average number of assessed days was not significantly different between clinical groups. The average number of assessments per day decreased over the course of the study. | *Reliability:* Not reported.    *Validity:* Not reported. |
| 40 | Janssens *et al.* (2012) | Patients [psychotic disorders]; healthy controls | Random | 10 | 6 days | Yes (behaviour) | Yes | *Compliance:* 73% (controls), 65% (patients). | *Reliability:* Not reported.  *Validity:* Referred to the literature. |
| 41 | Jappe *et al.* (2014) | Patients [AN; EDNOS-AN] | Hybrid (semi-random, event-contingent, interval-contingent) | 6 | 14 days | Yes (stress) | Yes | *Compliance:* 86% (group 1) and 87% (group 2).  End of day report compliance was 90% and 89%. | *Reliability:* Not reported.  *Validity:* Referred to the literature that the ESM questionnaire has shown convergent validity with endocrine stress measures. |
| 42 | Johns *et al.* (2019) | Patients [bipolar disorder; MDD]; healthy controls | Semi-random | 4 | 14 days | Yes (PA, NA, cognitive states) | Yes | Compliance: 82.6%.  Compliance was not different across diagnostic groups. | *Reliability:* Not reported.  *Validity:* Not reported. |
| 43 | Karr *et al.* (2013) | Patients [BN] | Hybrid (semi-random, event-contingent, interval-contingent) | 6 | 14 days | Yes (NA, PA, behaviour) | Yes | Not reported for semi-random prompts. | *Reliability:* Internal consistency reported for NA (Cronbach α=.92), and for PA (Cronbach α=.91).  *Validity:* Not reported. |
| 44 | Khazanov *et al.* (2019) | Patients [MDD, GAD]; healthy controls | Semi-random | 8 | 6 days | Yes, (PA, NA, cognition, behaviour, symptoms, | No | Compliance: 72%.  Two (out of 151) participants’ data were lost due to technical problems, 1 participant did not return the electronic diary, and 3 participants withdrew due to time conflicts. | *Reliability:* Items were averaged at each time point to form momentary positive affect scale (within-person ω = .63 to.72) and negative affect scale (ω = .75 to .77). Momentary worry scale (within-person α = .84 to .85) and rumination (α =.82 to .85) variables.  *Validity:* Momentary NA and PA correlated highly with trait NA or trait PA, respectively, on the PANAS (.60 to .61). Momentary worry correlated highly with trait worry assessed by the Penn State Worry Questionnaire (.58 to .59). Momentary rumination correlated highly with trait rumination assessed by the Ruminative Responses Scale. |
| 45 | Kimhy *et al.* (2014) | Patients [schizophrenia]; healthy controls | Semi-random | 10 | 2 days | Yes (PA,NA) | Yes | *Compliance:* 80% (patients) and 85% (controls). | *Reliability:* Not reported.    *Validity:* Emotional Granularity Indices were calculated by correlating ESM items. |
| 46 | Klippel *et al.* (2018) | Patients [psychosis], high-risk [first-degree relatives], healthy controls | Semi-random | 10 | 6 days | Yes (NA, stress, psychotic experiences) | No | Not reported (only criteria for data inclusion). | *Reliability:* Not reported.  *Validity:* Referred to prior literature. |
| 47 | Klippel *et al.* (2017) | Patients [psychosis]; high-risk population [psychosis]; healthy controls | Semi-random | 10 | 6 days | Yes (PA, NA, stress) | Yes | Not reported.  15 (out of 165) participants were excluded due to insufficient number of valid responses (≤19). | *Reliability:* Internal consistency was reported for psychotic experience (Cronbach α=.90).  *Validity:* Good concurrent validity of psychotic experiences with negative affect (r=.68). |
| 48 | Kuepper *et al.* (2013) | Patients [psychosis]; healthy controls | Semi-random | 12 | 6 days | Yes (NA, PA) | Yes | *Compliance*: 44% (patients) and 60% (controls).  31% of participants were excluded (< 24 valid reports). | *Reliability:* Internal consistency reported for PA (Cronbach α=.88); NA (Cronbach α=.82); paranoia (Cronbach α=.72), and hallucinations (Cronbach α=.84).  *Validity:* Not reported. |
| 49 | Lataster *et al.* (2011) | Patients [psychotic disorder] | Random | 10 | 6 days | Yes (PA and NA) | Yes | Not reported. | *Reliability*: Internal consistency was reported for PA (Cronbach α=.85), for NA (Cronbach α=.83), for hallucination (Cronbach α=.74), and for delusion (Cronbach α=.66).  *Validity:* Not reported. |
| 50 | Lavender *et al.* (2013a) | Patients [AN; EDNOS-AN] | Hybrid (semi-random, event-contingent, interval-contingent) | 6 | 14 days | Yes (NA) | Yes | *Compliance:* 87%.  Compliance with end-of-day rating: 90%. | *Reliability:* Internal consistency reported for NA (Cronbach α=.94) and anxiety (Cronbach α=.92).  *Validity:* Significant correlations of ESM affective liability or ESM anxiousness and respective validated questionnaires. |
| 51 | Lavender *et al.* (2013b) | Patients [AN; EDNOS-AN] | Hybrid (semi-random, event-contingent, interval-contingent) | 6 | 14 days | Yes (NA) | Yes | *Compliance:* 87%.  Compliance with end-of-day rating: 90%. | *Reliability:* Internal consistency reported for anxiety (Cronbach α=.92).  *Validity:* Not reported. |
| 52 | Lavender *et al.* (2013c) | Patients [AN; EDNOS-AN] | Hybrid (semi-random, event-contingent, interval-contingent) | 6 | 14 days | Yes (eating behaviour) | Yes | *Compliance:* 87%.  Compliance with end-of-day rating: 89%. | *Reliability:* Not reported.  *Validity:* Not reported. |
| 53 | Lavender *et al.* (2013d) | Patients [AN; EDNOS-AN] | Hybrid (semi-random, event-contingent, interval-contingent) | 6 | 14 days | Yes (NA, PA) | Yes | *Compliance:* 87%. | *Reliability:* Internal consistency reported for NA (Cronbach α=.94), PA (Cronbach α=.92), momentary actual-ideal self-discrepancy (Cronbach α=.86), and actual-ought self-discrepancy (Cronbach α=.83).  *Validity:* Not reported. |
| 54 | Lavender et al., (2016) | Patients [AN; EDNOS-AN] | Hybrid (semi-random + interval contingent, event-contingent) | 6 | 14 days | Yes (PA, NA, mood) | No | *Compliance*: 87%  Compliance for end of day recordings: 89%. | *Reliability:* Internal consistency was reported for PA (Cronbach α=.92), for NA (Cronbach α=.94) and for the profile of mood states (Cronbach α=.92).  *Validity*: Not reported. |
| 55 | Lavender *et al.* (2016) | Patients [AN] | Hybrid (semi-random, event-contingent, interval-contingent) | 6 | 14 days | Yes (NA, PA, behaviour) | Yes | *Compliance*: 87%  Compliance for end of day recordings: 89%. | *Reliability:* Not reported.  *Validity:* Not reported. |
| 56 | Leahey *et al.* (2011) | Patients [BN; EDNOS]; healthy controls | Semi-random | 6 | 5 days | Yes (self-esteem, appearance, PA, NA) | Yes | *Compliance:* Over 80%, but no actual number reported. Significant time effect: participants made more appearance comparisons at the beginning of the sampling phase | *Reliability:* Reference to the literature.  *Validity:* Not reported |
| 57 | Leendertse *et al.* (2018) | Patients [psychotic disorder]; healthy controls | Semi-random | 10 | 6 days | Yes (PA, NA, activity, quality of life) | Yes | Not reported.  4% of participants provided an insufficient number of ESM reports (< 20 valid reports). | *Reliability:* Not reported.  *Validity:* ESM measure of momentary quality of life has been validated in previous work. |
| 58 | Leraas *et al.* (2018) | Patients [BN] | Hybrid ( semi-random + interval-contingent, event-contingent) | 6 | 14 days | Yes (PA, NA) | Yes | Not reported. | *Reliability:* Internal consistency was reported for NA (Cronbach α=.92) and PA (α=.91).  *Validity:* To assess the external validity of the latent profiles, an ANOVA was used to compare the profiles of co-occurring diagnoses. |
| 59 | Le Grange *et al.* (2013) | Patients [AN; EDNOS-AN] | Hybrid (semi-random, event-contingent, interval-contingent) | 6 | 14 days | Yes (NA, PA, behaviour) | Yes | *Compliance*: 87%  Compliance for end of day recordings: 89%. | *Reliability:* Not reported.  *Validity:* Not reported. |
| 60 | Mackesy-Amiti and Donenberg (2020) | Patients [drug users] | Semi-random | 4-6 | 15 days | Yes (PA, NA) | Yes | *Compliance:* 44% | *Reliability:* Internal consistency was reported for PA (Cronbach α=.81) and NA (α=.91). The response format did not affect NA or PA intensity.  *Validity:* Not reported. |
| 61 | McKone *et al.* (2019) | Patients [ADHD] | Event-contingent | - | 10 days | Yes (drinking behavior, context) | Yes | Not reported. | *Reliability:* ICCs for outcomes ranged from .06 to .58, indicating that the sample provided sufficient variability. Post-drink reports were limited to a maximum of four drinks per episode to balance comprehensiveness and accuracy.  *Validity:* Not reported. |
| 62 | Mason *et al.* (2017) | patients [BN] | Semi-random | 6 | 14 days | Yes (NA) | Yes | *Compliance:* 86% | *Reliability:* Internal consistency was reported for NA (Cronbach α=.92) and dissociation (Cronbach α=.83).  *Validity:* not reported. |
| 63 | Mason *et al.* (2018) | Patients [AN] | Hybrid (semi-random + interval-contingent) | 6 | 14 days | Yes (stress, anxiety, eating behaviour) | No | *Compliance*: 87%. | *Reliability:* Inter-item-correlations between the three stress items were r=.43, .63, and .64, suggesting consistency without excessive overlap. The anxiety scale showed high internal consistency (Cronbach α=.92).  *Validity:* Not reported. |
| 64 | Muehlenkamp *et al.* (2009) | patients [BN] | Hybrid (semi-random, event-contingent, interval-contingent) | 6 | 14 days | Yes (PA, NA, behaviour) | Yes | Not reported.  10 out of 143 participants provided incomplete EMA data and their data was excluded. | *Reliability:* Internal consistency was reported for NA (Cronbach α=. 92) and PA (Cronbach α=.91).  *Validity:* Not reported. |
| 65 | Oorschot *et al.* (2012) | Patients [schizophrenia spectrum disorders]; healthy controls | Random | 10 | 6 days | Yes (PA, NA, hallucinations) | Yes | Compliance: 65% to 80% | *Reliability:* Internal consistency was reported for PA (Cronbach α=.84), for NA (Cronbach α=.83) and for hallucinations (Cronbach α=.66).  *Validity:* Not reported. |
| 66 | Oorschot *et al.* (2013) | Patients [schizophrenia; [schizoaffective disorder]; healthy controls | Random | 10 | 6 days | Yes (NA, PA) | Yes | *Compliance:* 70% (low negative symptom patients), 63 % (high negative symptom patients) and 80% (controls).  12 (of 334) participants were excluded due to less than 20 beeps. | *Reliability:* Internal consistency was reported for NA (Cronbach α=.82), and PA (Cronbach α=.81).  *Validity:* Not reported. |
| 67 | Pearson *et al.* (2017) | Patients [BN] | Semi-random | 6 | 14 days | Yes (NA, stress) | Yes | *Compliance:* 86% | *Reliability:* High internal consistency of NA scale (Cronbach α=.92).  *Validity:* Not reported. |
| 68 | Pearson *et al.* (2016) | Patients [BN] | Hybrid ( semi-random, interval-contingent, event-contingent) | 6 | 14 days | Yes (NA, eating behaviour) | Yes | Not reported. | *Reliability:* Not reported.  *Validity:* Not reported. |
| 69 | Peerbooms *et al.* (2012) | Patients [psychotic disorder]; healthy controls | Random | 10 | 6 days | Yes (stress, psychotic experiences) | Yes | *Compliance:* 73%.  Two (out of 118) controls and 5 (out of 98) patients were excluded due to < 20 answered prompts. | *Reliability:* Internal consistency was reported for psychotic experiences (Cronbach α=.71).  *Validity:* Not reported. |
| 70 | Pinkham *et al.* (2020) | Patients [schizophrenia; schizoaffective disorder; bipolar disorder; MDD] | Not reported | 3 | Study 1: 30 days Study 2: 10 days | Yes (PA, NA, activity, stress) | Yes | *Compliance:* 78 % (study 1) and 84 % (study 2). | *Reliability:* Not reported.  *Validity:* Stability of ESM scores over time (pre- and post-pandemic) was investigated (correlation). |
| 71 | Pisetsky *et al.* (2016) | Patients [BN] | Hybrid (semi-random, event-contingent, interval-contingent) | 6 | 14 days | Yes (NA, PA) | Yes | Not reported for random prompts. | *Reliability:* Internal consistency was reported for the four abbreviated lower-order NA subscales (Cronbach α=.80, .89, .79 and .81).  *Validity:* CFA of NA scale was conducted and replicated a four factor structure. However, disgust loaded on the hostility factor. |
| 72 | Reininghaus *et al.* (2016a) | Patients [psychosis]; high-risk population [psychosis]; healthy controls | Semi-random | 10 | 6 days | Yes (NA, stress) | Yes | Not reported (only criteria for data inclusion). | *Reliability:* Internal consistency was reported for NA (Cronbach α=.86), and social stress (Cronbach α=.62).  *Validity:* Good concurrent validity of ESM measures of NA and psychotic experiences, as well as ESM threat anticipation item with anxious mood. The item on outsider status correlated with ESM social and area-related stress measures indicating the item taps a distinct but related aspect of social stress. |
| 73 | Reininghaus *et al.* (2016b) | Patients [psychosis]; high-risk population [psychosis]; healthy controls | Semi-random | 10 | 6 days | Yes (NA, stress) | Yes | *Compliance:* 91%. | *Reliability:* Not reported.  *Validity:* Referred to a previous study with this sample demonstrating the feasibility, reliability, and validity of the approach. |
| 74 | Rintala *et al.* (2019) | Patients [psychosis, MDD]; at-risk [first-degree relatives]; high-risk [psychometric risk for psychosis] | Semi-random | 10 | 4-6 days | No | Yes | *Compliance:* 78%.  Compliance declined across days, reaching a low on the 5th day with 73% and varied significantly across assessments depending on the time within a day, with highest compliance between 12 p.m. and 1.30 p.m. (83) and lowest compliance between 7.30 a.m. and 9 a.m. (56%). | *Reliability:* Referred to prior literature.  *Validity:* Not reported. |
| 75 | Ruscio *et al.* (2015) | Patients [MDD; GAD]; healthy controls | Semi-random | 8 | 7 days | Yes (PA, NA, rumination) | No | *Compliance*: 72%.  The number of assessments completed did not differ by diagnostic group. | *Reliability:* Internal consistency was reported for PA (Cronbach α=.8), for NA (Cronbach α=.8) and for rumination (Cronbach α=.9).  *Validity:* Validity of the ESM rumination scale, negative affect and positive affect scale were supported by strong associations with questionnaires (r =.57 to .62). |
| 76 | Rusby *et al.* (2019) | At-risk population [youths] | Random | 18 in total | 4 days (10 times) | Yes (PA, NA) | No | *Compliance:* 76%.  Retention at each wave of assessment: 92% to 73%. | *Reliability:* Not reported.  *Validity:* Not reported. |
| 77 | Schaefer *et al.* (2020) | Patients [binge eating disorder] | Hybrid (semi-random, interval-contingent) | 5 | 7 days | Yes (PA, NA, eating behaviour) | No | *Compliance*: 76 %.  No interaction between compliance group status and temporal effects | *Reliability:* Internal consistency reported for NA (Cronbach α=.80), PA (Cronbach α=.88) and negative affect facet scales (ranged from α=.81 to α=.91).  *Validity:* Perceived loss of control during eating episodes represents a defining feature of binge-eating episodes underlying construct validity of ESM assessments. |
| 78 | Schreiber-Gregory *et al.* (2013) | Patients [binge eating disorder] | Hybrid (semi-random, interval-contingent) | 7 | 14 days | Yes (eating behaviour) | Yes | Not reported. | *Reliability:* Not reported.  *Validity:* Not reported. |
| 79 | Scott *et al.* (2017) | Individuals at-risk from general population [BPD] | Semi-random | 6 | 21 days | Yes (NA, behaviour) | Yes | *Compliance*: 76% (mean); 85% (median) | *Reliability:* Between- / within-person internal constancies were reported for NA (Cronbach α=.98 / α=.71), anger (Cronbach α=.98 / α=.85), and shame (Cronbach α=.98 / α=.83).  *Validity:* Demonstrating the construct validity of the ESM aggression measure, individuals with high trait questionnaire scores, had higher momentary aggression. |
| 80 | Selby *et al.* (2012) | Patients [BN; BPD] | Hybrid (semi-random, event-contingent) | 6 | 14 days | Yes (NA, PA, behaviour) | Yes | Not reported.  75% of responses initiated recording within 20 minutes of being signaled, with an average response time of 12 minutes. | *Reliability:* Internal consistency was reported for NA (Cronbach α=.92), and PA (Cronbach α=.91).  *Validity:* Not reported. |
| 81 | Serre *et al.* (2018) | Patients [SUD] | Fixed | 4 | 14 days | Yes (NA, PA, behaviour) | No | *Compliance*: 80 % (cannabis use disorder), 82% (alcohol use disorder), 83% (opiate users), and 88% (tobacco addicts).  Due to a restricted number of assessments occurring on the starting and ending day of the study, only data collected between days 2 and 13 were analyzed. | *Reliability:* Not reported.  *Validity:* Referred to the literature. |
| 82 | Simons *et al.* (2015) | Patients [MDD] | Semi-random | 10 | 3 days (six times) | Yes (PA, NA, activity, context) | Yes | Not reported.  10 (out of 102) participants partially responded to prompts or withdrew from the study. | *Reliability:* Not reported.  *Validity:* Not reported. |
| 83 | Smyth *et al.* (2009) | patients [BN] | Hybrid (semi-random, event-contingent, interval-contingent) | 6 | 14 days | Yes (PA, NA, stress, behaviour) | Yes | Not reported.  10 out of 143 participants completed the ESM assessment. | *Reliability:* Internal consistency was reported for the anger-hostility subscale (Cronbach α=.89), PA (Cronbach α=.91) and NA (Cronbach α=.92).  *Validity:* Not reported. |
| 84 | Solhan *et al.* (2009) | Patients [BPD; MDD/ dysthymia] | Semi-random | 6 | 28 days | Yes (NA) | Yes | *Compliance: 9*2%. | *Reliability:* Not reported.  *Validity:* Modest correlation between EMA measure and trait questionnaire. |
| 85 | Sperry *et al.* (2020) | Individuals at-risk from general population [hypomanic personality] | Semi-random | 8 | 7 days, 2 times | Yes (PA, NA) | No | Not reported.  138 out of 147 participants had sufficient number of completed reports at baseline. 77% of participants were assessed at follow-up. | *Reliability:* The NA index showed good within-person reliability (ω=0.74) and excellent between-person reliability (ω= 0.93). The PA index showed good within-person reliability (ω=0.70) and excellent between-person reliability (ω=0.92).  *Predictive Validity:* Within person variance was the most robust predictor of psychopathology at follow up over and above other emotion dynamics. |
| 86 | Tasca *et al.* (2009) | Patients [AN; BN] | Hybrid (semi-random, event-contingent, interval-contingent) | 3 | 7 days | Yes (eating behaviour) | Yes | Not reported. | *Reliability:* Not reported.  *Construct validity* reported based on Exploratory Factor Analysis. |
| 87 | Thewissen *et al.* (2008) | Patients [psychotic disorder]; individuals at-risk from general population [psychosis] | Random | 10 | 6 days | Yes (symptoms) | No | *Compliance:* 74%.  Exclusion of participants with less than 20 filled reports. | *Reliability:* Internal consistency reported for paranoia (Cronbach α=.89), and for self-esteem (Cronbach α=.84).  *Construct validity:* PCA revealed one factor for momentary paranoia which correlated with the PANSS interview rating and paranoia scale. PCA revealed one factor for self-esteem. |
| 88 | Thewissen *et al.* (2011) | Patients [psychotic disorder]; individuals at-risk from general population [psychosis] | Semi-random | 10 | 6 days | Yes (NA, self-esteem, psychotic experiences) | Yes | *Compliance:* 72%.  Twenty-two (out of 183) participants terminated the study prematurely, due to severity of psychotic symptoms (N = 11), cognitive incapability (N = 5), or lack of cooperation (N = 6) and 3 were excluded due to insufficient number of observations. | *Reliability:* Internal consistency was reported for the self-esteem scale (Cronbach α=.84), for paranoia (Cronbach α=.90) and hallucination (Cronbach α=.74).  *Validity:* Factor analysis of self-esteem items showed one factor accounting for 68% of the total variance. Factor analysis of paranoia items revealed one factor accounting for 78% of the total variance and for the hallucination scale one factor explaining 84% of the total variance. |
| 89 | Udachina *et al.* (2017) | Patients [SSD] | Semi-random | 10 | 6 days | Yes (PA, NA, symptoms) | Yes | *Compliance:* 65% (patients), 82% (controls).  16% of participants did not return the booklets and/or failed to comply with the protocol. 15% of data obtained was excluded, as participants completed <20 reports. | *Reliability:* Internal consistency was reported for psychotic experiences (Cronbach α=.94), deservedness (Cronbach α=.90), NA (Cronbach α=.87), PA (Cronbach α=.77) and self-esteem (Cronbach α=.81).  *Validity:* A PCA revealed one factor for psychotic experiences and one self-esteem. PCA identified 2 factors with eigenvalues >1 and explaining 66% of the total variance, thus two factor-based scales (positive and negative affect) were created. |
| 90 | Wichers *et al.* (2007) | Patients [MDD]; high-risk [relatives, i.e. twin siblings] | Random | 10 | 5 days | Yes (NA, stress) | Yes | Not reported.  31 (out of 621) participants were excluded as they answered < 30% prompts. | *Reliability:* Internal consistency reported for NA (Cronbach α=.76).  *Validity:* Not reported. |
| 91 | Wigman *et al.* (2015) | Patients [MDD; psychotic disorder]; healthy controls | Semi-random | 10 | 5-6 days | Yes (mental states) | No | Not reported | *Reliability:* Not reported.  *Validity:* Correlations of all mental states were moderate to substantial, ranging between 0.47 and 0.83. |
| 92 | Williams-Kerver and Crowther (2020) | High-risk population [eating disorders] | Hybrid (semi-random, event-contingent, interval-contingent) | 4 | 7 days | Yes (PA, NA, eating behaviour) | No | *Compliances:* 79%.  A least 20% of prompts had to be completed. | *Reliability:* Not reported.  *Validity:* Referred to literature and argued that validity of affect ESM items is evident from their unique position in a multitude of two-dimensional models. |
| 93 | Wonderlich *et al.* (2007) | Patients [BN] | Hybrid (semi-random, event-contingent, interval-contingent) | 6 | 14 days | Yes (eating behaviour) | Yes | *Compliance:* 92%.  Seven (of 142) participants provided incomplete ESM data. | *Reliability:* Internal consistency reported of profile of mood states (Cronbach α=.89).  *Validity:* Not reported. |
| 94 | Wonderlich *et al.* (2015) | Patients [AN; binge eating disorder; obesity] | Hybrid ( semi-random + interval-contingent, event-contingent) | 6 | 14 days | Yes (NA, eating behaviour) | Yes | Not reported.  Sample 1: Three (out of 121) participants had ESM compliance rates of less than 50% and were excluded from analyses.  Sample 2: 10 (out of 143) participants provided incomplete data in the ESM. | *Reliability:* Internal consistency was reported for NA in the 3 samples (Cronbach α ranged from .91 to .94).  *Validity:* Correlations between retrospective measures and the ESM measures were ranging from r= 0.50 to r= 0.78. |
| 95 | Yaroslavsky *et al.* (2019) | Patients [MDD; dysthymia, personality disorder, anxiety, PTSD, OCD]; at-risk [elevated levels at borderline personality scale]; controls | Semi-random | 5 | 7 days | Yes (NA) | No | *Compliance:* 78%.  Less than 5% of ESM data were missing. | *Reliability:* Items were aggregated to form immediate NA (Cronbach α=.83) and peak  NA (Cronbach α=.66) indices that evidenced acceptable internal consistency. Context information was coded by independent raters (Inter-rater reliability: κ = 0.98).  *Validity:* Not reported. |
| 96 | Zunker *et al.* (2011) | Patients [BN] | Hybrid (semi-random + interval-contingent, event-contingent) | 6 | 14 days | Yes (eating behavior) | Yes | Not reported. | *Reliability:* Not reported.    *Validity:* Not reported. |
| **Telephone calls /emails /online screenings** | | | | | | | | | |
| 97 | Aikens *et al.* (2015) | Patients [MDD] | Not reported | Weekly | 26 weeks | Yes (symptoms) | Yes | *Compliance*: 72%. | *Reliability:* Not reported.  *Validity:* Not reported. |
| 98 | Bilderbeck *et al.* (2016) | Patients [bipolar disorder] | Not reported | Weekly | 12 months | No (symptoms) | No | *Compliance:* 77 % and 71%. | *Reliability:* Not reported.  *Validity:* Not reported. |
| 99 | Duffy *et al.* (2019) | High-risk [offspring of bipolar parents]; healthy controls | Not reported | Daily or weekly | 90 days | Yes (mood) | Yes | *Compliance in the first 30 days*: 78% (high-risk) and 97% (controls).  Compliance over 90 days: 56% (high‐risk) and 69% (controls). | *Reliability*: Not reported.  *Validity:* Not reported. |
| 100 | Fitzsimmons-Craft *et al.* (2019) | Individuals at-risk [eating disorder] | Not reported | Daily | 24 months | Yes (eating behaviour) | Yes | *Compliance*: 86% | *Reliability:* Not reported.  *Validity*: Not reported. |
| 101 | Kordy *et al.* (2016) | Patients [MDD] | Not reported | Every 14 days | 24 months | Yes (symptoms) | Yes | *Compliance*: 69% | *Reliability:* Not reported.  *Validity:* Not reported. |
| 102 | Morgan *et al.* (2017) | Patients [anxiety disorders]; healthy controls | Semi-random | 2-4 | 5 days (5 times) | Yes (PA, context) | Yes | There were no group differences in EMA compliance, with socially anxious youth completing 57.07/70 possible calls (82%), other anxious youth completing 53.71/70 possible calls (77%), and healthy youth completing 54.26/70 (78%) possible calls | *Reliability:* Momentary ratings of happy affect were highly correlated with momentary ratings of cheerful, excited, and interested affect.  *Validity:* Interviews were used to cross-validate ESM. |
| 103 | Perez Arribas *et al.* (2018) | Patients [bipolar disorder; BPD]; healthy controls | Not reported | Daily | 90 days | Yes (PA, NA) | Yes | *Compliance*: 81%.  9 (of 139) participants who withdrew consent or failed to provide at least 2 months of data were excluded. | *Reliability:* Not reported.  *Validity:* Not reported. |
| 104 | Primack *et al.* (2011) | Patients [MDD]; healthy controls | Not reported | 12 calls per weekend | 5 weekends (over the course of 8 weeks) | Yes (behaviour) | Yes | *Compliance*: 89% | *Reliability*: Not reported.  *Validity:* Not reported. |
| 105 | Scott *et al.* (2015) | Individuals at-risk from general population [BPD] | Not reported | 4 | 7 days | Yes (behaviour) | Yes | *Compliance*: 86%. Data were excluded from three girls who completed less than seven calls over the course of the week. | *Reliability:* Not reported.  *Validity:* Not reported. |
| 106 | Schlam *et al.* (2020) | Smokers (nicotine) at risk for substance use | Not reported | 3 | 7 days (2 times) | Yes (NA) | Yes | Not reported | *Reliability:* Not reported.  *Validity:* Not reported. |
| 107 | Silk *et al.* (2018) | Patients [anxiety disorders] | Not reported | 2 to 3 | 5 days | Yes (PA,NA) | Yes | *Compliance*: 98% | *Reliability:* Not reported.  *Validity:* Not reported. |
| 108 | Tsanas *et al.* (2016) | Patients [bipolar disorder; BPD]; healthy controls | Not reported | Daily and weekly | 3 months to 12 months | Yes (mood) | Yes | *Compliance:* 81% for weekly assessment, 86% for daily assessment. | *Reliability:* Not reported.  *Validity:* Validity of the daily questionnaire was confirmed by satisfactory correlation with standard scales. |

*Note:* ^1^ Data aggregated refers to the calculation of a mean over the assessment period. ADHD: Attention Deficit Hyper Disorder; AN: Anorexia nervosa; AUD: Alcohol Use Disorder; GAD: Generalized Anxiety Disorder; BN: Bulimia Nervosa; BPD: Borderline Personality Disorder; ESM: Experience Sampling Methodology; MDD: Major Depressive Disorder; SSD: Schizophrenia Spectrum Disorders; OCD: Obsessive Compulsive Disorder; EDNOS-AN: eating disorder not otherwise specified; ICC: Intra-Class Correlation; PCA: Principal components analysis; PA: positive affect, NA: negative affect, mean compliance for ESM studies: 79%, mean compliance for studies collecting data via Telephone calls /emails /online screenings: 83%.

# **Table S3. Summary of included studies using sensors.**

| **Number** | **Reference** | **Population** | **Sensor type /**  **Sampling** | **Prompts per day / sampling frequency** | **Assessment period** | **Primary outcome is a time intensive measure** | **Data aggregated^1^** | **Compliance and data quality** | **Psychometric properties (Reliability,**  **Validity)** |
| --- | --- | --- | --- | --- | --- | --- | --- | --- | --- |
| 1 | Baerg *et al.* (2011) | Patients [developmental coordination disorder and ADHD]; healthy controls | Accelerometry at waist / continuous | 30-s epochs | 7 days | Yes (physical activity) | Yes | Not reported. | *Reliability:* Not reported.  *Validity:* Not reported. |
| 2 | Banihashemi *et al.* (2016) | Patients [affective disorders]; healthy controls | Actigraphy / continuous | 30-s or 1-min epochs | 5 to 22 days | Yes (physical activity) | No | Not reported | *Reliability:* Not reported.  *Validity:* Not reported. |
| 3 | Benard *et al.* (2019) | Patients [BPD]; healthy controls | Actigraphy at non-dominant wrist / continuous | 1-min epochs | 21 days | Yes (sleep) | Yes | Not reported | *Reliability:* Not reported.  *Validity:* Not reported. |
| 4 | Bergwerff *et al.* (2016) | Patients [ADHD); healthy controls | Actigraphy at non-dominant wrist/ continuous | 15-s epochs | 3 days | Yes (sleep) | Yes | Only data derived from nights prior to school days were used to control for shifts in sleep patterns during weekends. Data was available from 48 (out of 124) participants for 3 school nights, and of 42 (out of 124) participants of two school nights and of 34 (out of 124) participants for one school night. | *Reliability:* The use of data from one to three nights was mentioned as a limitation, as commonly data collection from 5 nights is recommended in terms of reliability.  *Validity:* Not reported. |
| 5 | Blytt *et al.* (2018) | Patients [dementia; MDD] | Actigraphy at dominant wrist / continuous | 1-min epochs | 14 days | Yes (sleep) | Yes | Not reported. | *Reliability:* Not reported.  *Validity:* Not reported. |
| 6 | Bracht *et al.* (2012) | Patients [schizophrenia; schizophreniform disorder] | Actigraphy at non-dominant hand/ continuous | 2-s epochs | 24h | Yes (physical  activity) | Yes | Not reported.  Nocturnal activity was excluded. | *Reliability:* Inter-rater reliability of scale was reported to be satisfactory.  *Validity:* Study aimed to validate the Bern Psychopathology Scale by using objective measure of motor behavior. |
| 7 | Bratland-Sanda *et al.* (2011) | Patients [eating disorder]; healthy controls | Actigraphy at hip / continuous | Not reported | 7 days | Yes (physical activity) | Yes | Not reported.  Nocturnal activity and sequences >10 min of consecutive zero counts were excluded. | *Reliability:* Not reported.    *Validity:* Referred to other studies that validated the actigraphy sensor. |
| 8 | Chen *et al.* (2016) | Patients [schizophrenia]; healthy controls | Actigraphy at non-dominant wrist / continuous | Not reported | 7 days | Yes (physical activity) | Yes | Participants were excluded if the accelerometers were not worn for at least 10 h per day (excluding sleep period) for at least 5 days (n = 38 out of 199). The mean wear time was 13.9 (S.D. = 2.1) h per day for the schizophrenia group and 16.3 (S.D. = 1.4) h per day for the healthy controls. | *Reliability:* Not reported.  *Validity:* Not reported. |
| 9 | Choi *et al.* (2019) | Patients [MDD]; healthy controls | Accelerometry at wrist/ continuous | Not reported | 3 days | Yes (physical activity) | Yes | Not reported. | *Reliability:* Not reported.  *Validity:* Not reported. |
|  |  |  |  |  |  |  |  |  |  |
| 10 | Difrancesco *et al.* (2019) | Patients [MDD; anxiety]; healthy controls | Actigraphy at dominant wrist / continuous | 1-min epochs | 14 days | Yes (sleep) | Yes | Not reported.  Data were included in the analyses if at least 1 week day and 1 weekend day of data was available, with at least 16h recorded per day and per night. | *Reliability*: Not reported.  *Validity:* Expected between-group differences in movement patterns were found and interpreted as a criterion for the ecological validity of the actigraphy measure. |
| 11 | Duncan *et al.* (2017) | Patients [schizophrenia, schizoaffective disorder] | Actigraphy at hip / continuous | Not reported | 7 days | Yes (physical activity) | Yes | Data-quality was considered acceptable when accelerometer recorded at least 600 min wear time per day, for at least four days within seven days. For a week: at least four days of the 7-day wear period were required. | *Reliability:* Not reported.  *Construct validity:* There was agreement between accelerometry and the 24 hour recall of physical activity. |
| 12 | Faedda *et al.* (2016) | Patients [bipolar disorder, ADHD; MDD]; healthy controls | Actigraphy at non-dominant wrist / continuous | 1-min epochs | 3-5 days | Yes (physical activity) | Yes | Not reported, only that actigraphy was easily obtained. | *Reliability:* Not reported.  *Validity:* Not reported. |
| 13 | Fang *et al.* (2016) | Patients [schizophrenia]; healthy controls | Actigraphy at non-dominant wrist / continuous | Not reported | 7 days | Yes (sleep) | Yes | Sleep indices had missing data for 26.6% in the original dataset which exceeded the acceptable criteria (< 10%). | *Reliability:* Not reported.  *Validity:* Not reported. |
| 14 | Goodlin-Jones *et al.* (2009) | Patients [autism, developmental delay without autism]; healthy controls | Actigraphy at non-dominant wrist / continuous | Not reported | 7 days | Yes (sleep) | Yes | Not reported. | *Reliability:* Not reported.  *Validity:* Not reported. |
| 15 | Gregory *et al.* (2011) | Patients [anxiety disorders; MDD]; healthy controls | Actigraphy / continuous | 1-min epochs | 7 days | Yes (sleep) | Yes | Not reported. | *Reliability:* Not reported.  *Validity:* Child  Behavior Checklist sleep items validated based on objective measures of sleep. |
| 16 | Hensch *et al.* (2019) | Individuals at-risk from general population [BPD] | Actigraphy / continuous | Not reported | 5 to 7 days | Yes (sleep) | Yes | Not reported. | *Reliability:* Not reported.  *Validity:* Not reported. |
| 17 | Keyes *et al.* (2015) | Patients [AN, GAD]; healthy controls | Actigraphy / continuous | Not reported | 7 days | Yes (physical activity) | Yes | *Compliance:* 41% (healthy controls), 44% (AN-inpatients), 47% (GAD), 62%, AN (out-patients)  (criteria: worn for at least 6 days and for at least 3 consecutive hours each day) | *Reliability:* Not reported.  *Validity:* Not reported. |
| 18 | McKenna *et al.* (2014) | Patients [bipolar disorder]; healthy controls | Actigraphy at non-dominant wrist / continuous | 1-min epochs | 7 days | Yes (sleep) | Yes | Not reported. | *Reliability:* Not reported.  *Validity:* Not reported. |
| 19 | Pagani *et al.* (2016)) | Patients [bipolar disorder]; at-risk [relatives of bipolar patients] | Actigraphy at dominant wrist / continuous | 1-min epochs | 14 days | Yes (physical activity) | Yes | Not reported.  80 recordings (out of 636) were excluded (due to data quality (42), device failures (8), participant illness (10), irregular schedule (14), mood crisis (6)). | *Reliability:* Not reported.  *Validity:* Not reported. |
| 20 | Piette *et al.* (2011a) | Patients [type 2 diabetes and MDD] | Pedometry/ continuous | During waking hours | 7 days | Yes (physical activity) | Yes | Not reported. | *Reliability:* Not reported.  *Validity:* Not reported. |
| 21 | Piette *et al.* (2011b) | Patients [diabetes and MDD] | Pedometry/ continuous | During waking hours | 7 days | Yes (physical activity) | Yes | Not reported. | *Reliability:* Not reported.  *Validity:* Not reported. |
| 22 | Ranum *et al.* (2019) | Individuals from general population at-risk [stratified for emotional and behavioral problems] | Actigraphy / continuous | Not reported | 7 days | Yes (sleep) | Yes | *Compliance:* 100% on at least 1 day. | *Reliability:* Not reported.  *Validity:* Not reported. |
| 23 | Sauchelli *et al.* (2015) | Patients [AN]; healthy controls | Accelerometry / continuous | 1-min epochs | 6 days | Yes (physical activity) | Yes | Not reported. | *Reliability:* Not reported.  *Validity:* Not reported. |
| 24 | Shou *et al.* (2017) | Patients [bipolar disorder,MDD]; High-risk [relatives of patients]; controls | Actigraphy at non-dominant wrist / continuous | 1-min epochs | 14 days | Yes (physical activity) | No | Not reported.  Missing data within a day: 18.4%, average length of consecutive missing interval was ~2 h. | *Reliability:* Not reported.  *Validity:* Not reported. |
|  |  |  |  |  |  |  |  |  |  |
| 25 | Smagula *et al.* (2018) | Patients [seasonal affective disorder]; healthy controls | Actigraphy / Not reported | Not reported | 7 to 14 days | Yes (physical activity) | No | Not reported.  Adequate actigraphy data (defined as at least 3 days of recording) was available from 70 (out of 78) participants at time point 1 and from 84 (out of 90) participants at time point 2. | *Reliability:* Not reported.  *Validity:* Not reported. |
| 26 | Stubbs *et al.* (2017) | Patients [schizophrenia]; healthy controls | Actigraphy at non-dominant wrist / continuous | Not reported | 7 days | Yes (physical activity) | Yes | Not reported. | *Reliability:* Not reported.  *Validity:* Not reported. |
| 27 | Walther *et al.* (2014) | Patients [psychosis spectrum disorder] | Actigraphy / continuous | 2-sec epochs | 24 h | Yes (physical activity) | No | *Compliance:* 88 % (between 10:00–11:00 am) and 95% between 3:00–4:00 pm. | *Reliability:* Not reported.  *Validity:* Not reported. |
| 28 | Wichniak *et al.* (2011) | Patients [schizophrenia spectrum disorder]; healthy controls | Actigraphy at wirst/ continuous | 10-s epochs | 7 days | Yes (sleep) | Yes | Not reported.  Minimal wear time of actigraphy was 21h/ day. | *Reliability:* Not reported.  *Validity:* Not reported. |
| 29 | Zebin et al., (2019) | Patients [severe mental illness]; healthy controls | Actigraphy at wrist / continuous | 100 Hz | 7 days | Yes (physical activity) | Yes | Not reported. | *Reliability:* Not reported.  *Validity:* Not reported. |

*Note:* ^1^ Data aggregated refers to the calculation of a mean over the assessment period. ADHD: Attention Deficit Hyper Disorder; AN: Anorexia nervosa; AUD: Alcohol Use Disorder; GAD: Generalized Anxiety Disorder; BPD: Borderline Personality Disorder; MDD: Major Depressive Disorder; OCD: Obsessive Compulsive Disorder; mean compliance in studies collecting sensor data: 82%.

# **Table S4****. Summary of included studies using mobile sensing.**

| **Number** | **Reference** | **Population** | **Data type** | **Prompts per day / sampling frequency** | **Assessment period** | **Primary outcome is a time intensive measure** | **Data aggregated^1^** | **Compliance and data quality** | **Psychometric properties (Reliability,**  **Validity)** |
| --- | --- | --- | --- | --- | --- | --- | --- | --- | --- |
| 1 | Birnbaum *et al.* (2017) | Patients [schizophrenia - self-disclosed]; healthy controls | Twitter timeline | Not applicable | Not reported | Yes (linguistic characteristics) | Yes | Not reported. | *Reliability:* Not reported.  *Validity:* Not reported. |
| 2 | Eichstaedt *et al.* (2018) | Patients [MDD]; individuals at-risk [help-seeking in an emergency department] | Social media usage | Not applicable | 6 years | Yes (linguistic characteristics) | Yes | Not reported. | *Reliability:* Not reported.  *Predictive validity:* Facebook language-based prediction models perform similarly to screening surveys in identifying patients with depression. |
| 3 | Fitzsimmons-Craft *et al.* (2019) | Individuals at-risk [eating disorder] | Online platform for screening and intervention | 24h epochs for symptom assessment | 2 years | Yes (restrictive eating) | Yes | Data included if at least 3 symptom reports available.  85.8% of participants engaged in intervention, 60.4% completed at least 2 sessions, average time spent in the intervention: 47 days | *Reliability:* Not reported.  *Validity:* Screening  algorithm was validated in other studies. |
| 4 | Friedmann *et al.* (2020) | Patients [PTSD]; high-risk population | GPS | 5-sec epochs when smartphone was moved | 7 days | Yes (physical activity) | No | 16% excluded due to entirely missing data (technical problems). | *Reliability:* Not reported.  *Validity:* Not reported. |
| 5 | Hswen *et al.* (2017) | Patients [schizophrenia - self-disclosed]; healthy controls | Twitter timeline | Not applicable | Not reported | Yes (linguistic characteristics) | Yes | Selection of target words did not include slang | *Reliability:* Not reported.    *Validity:* Not reported. |
| 6 | Hswen *et al.* (2018) | Patients [schizophrenia - self-disclosed]; healthy controls | Twitter timeline | Not applicable | 200 days | Yes (linguistic characteristics) | Yes | Not reported. | *Reliability*: Not reported.  *Validity*: Not reported. |
| 7 | Pratap *et al.* (2019) | Individuals at-risk from general population [psychometric risk: Patient Health Questionnaire and/or self-rated impairment social functioning) | ESM; Phone usage (mobility, phone usage logs, and missed calls) | ESM: daily mood ratings  Phone usage aggregated over 24h epochs | 12 weeks | Yes (mood) | Yes | Not reported. Participant was considered “active” during the week if any passive or active data were recorded at least once. No direct association between attrition and assessment incentives reported. Missing data was imputed using participant`s weekly median. | *Reliability:* Not reported.  *Validity:* Not reported. |
| 8 | Reece *et al.* (2017) | Patients [MDD, PTSD]; healthy controls | Twitter timeline | Not applicable | Twitter history up to most recent 3200 posts | Yes (twitter activity) | Yes | Applied quality  assurance measures in data collection process: MTurk workers who have completed at least 100 tasks, with a minimum 95% approval rating, have been found to provide reliable, valid survey responses | *Reliability:* Not reported.  *Validity:* Stratified five-fold cross-validation was used to optimize Random Forests hyper-parameters. |

*Note:* ^1^ Data aggregated refers to the calculation of a mean over the assessment period. MDD: Major Depressive Disorder; PTSD: Post Traumatic Stress Disorder; GPS: Global Positioning System;

**Table S5. Summary of studies using active and passive data acquisition methods combined**

| **Number** | **Reference** | **Population** | **Sampling scheme** | **Sampling frequency** | **Assessment period** | **Primary outcome is a time intensive measure** | **Data aggregated^1^** | **Measurement quality (compliance, % data)** | **Psychometric properties**  **(reliability, validity)** |  |
| --- | --- | --- | --- | --- | --- | --- | --- | --- | --- | --- |
| 1 | Blake *et al.* (2016) | Individuals at risk from general population [psychometric risk: high levels of anxiety and sleeping difficulties] | Diary^2^;  Actigraphy on non-dominant wrist / continuous | Daily;  Not reported | 7 days  (2 times, i.e. baseline and post-intervention) | Yes (sleep) | Yes | Not reported. | *Reliability:* Bedtimes and rest times were determined by visually screening the actograms using collective information of the actigraphy (algorithm/movement, light, event markers) and sleep diary.  *Validity:* To cross-validate the actigraphy variables, participants completed a paper sleep diary. However, no numbers were reported. |  |
| 2 | Blake *et al.* (2017) | Individuals at risk from general population [psychometric risk: high levels of anxiety and sleeping difficulties] | Diary^2^;  Actigraphy at wrist / continuous | Daily;  Not reported | 5 days | Yes (sleep) | Yes | Not reported. | *Reliability:* Not reported.  *Validity:* Not reported. |  |
| 3 | Blake *et al.* (2018) | Individuals at risk from general population [psychometric risk: high levels of anxiety and sleeping difficulties] | Diary^2^;  Actigraphy on non-dominant wrist / continuous | Daily;  Not reported | 6 days  (3 times, i.e., at baseline, post-intervention) | Yes (sleep) | Yes | *Compliance with sleep diary:* 75%  *Compliance with actigraphy:* 90%.  Missing data: 6.1% for actigraphy, 14.6% for sleep diary. Missing data was imputed.  Focused on sleep occurring on week days. | *Reliability:* Bedtimes and rest times were determined by visually screening the actograms using collective information of the actigraphy (algorithm/movement, light, event markers) and sleep diary.  *Validity:* Referred to the literature that visual inspection has a good correlation with polysomnography and is superior to automated actigraphy algorithms. | |
| 4 | Becker *et al.* (2019) | Patients [ADHD] | Diary^2^;  Actigraphy on non-dominant wrist / continuous | Daily;  1-min epochs | 14 days | Yes (sleep) | Yes | *Compliance with sleep diary:* 94% of participants provided data on 5 days.  *Compliance with actigraphy:* 60% of participants provided data on 5 days.  Data exclusion: Participants with <5 nights of weekday data and <3 nights of weekend data were excluded from analyses. | *Reliability:* Not reported.  *Validity:* Data were first validated using the wear-time sensor and a validation algorithm. Diaries were used to aid in adding sleep periods by verifying sleep and wake times. | |
| 5 | Chung *et al.* (2015) | Patients [insomnia and lifetime MDD] | Diary^2^;  Actigraphy on non-dominant wrist / continuous | Daily;  1-min epochs | 7 days  (3 times, i.e. baseline, post-intervention and 5-week follow-up) | Yes (sleep) | Yes | Not reported. | *Reliability:* Not reported.  *Validity:* Not reported. | |
| 6 | de Bruin *et al.* (2018) | Patients [insomnia] | Diary^2^;  Actigraphy at wrist/ continuous | Daily;  1-min epochs | 7 days | Yes (sleep) | Yes | At 2-month follow-up: 95 to 97% of participants provided data; at 6-month follow-up the range was between 44 to 59%. At 12-month follow-up compliance was 47%. | *Reliability:* Not reported.  *Validity:* Not reported. | |
| 7 | Geoffroy *et al.* (2019) | Patients [bipolar disorder] | Diary;  Actigraphy on non-dominant wrist / continuous | Daily;  1-min epochs | 21 days | Yes (sleep) | Yes | Not reported. | *Reliability:* Not reported.  *Validity:* Not reported. | |
| 8 | Hvolby *et al.* (2008) | Patients [ADHD, ODD, affective disorder, anxiety disorders OCD); healthy controls | Diary^2^;  Actigraphy at dominant wrist / continuous | Daily;  Not reported | 7 days/ 5 nights | Yes (sleep) | Yes | Not reported.  No missing data for passively assessed sleep variables. | *Reliability:* Not reported.  *Validity:* Not reported. | |
| 9 | Kaplan *et al.* (2019) | Patients [bipolar disorder] | Diary^2^;  Actigraphy on wrist / continuous | Daily;  Not reported | 14 days | Yes (sleep) | Yes | Compliance with sleep diary was confirmed in a subset of participants by calls to a voicemail. | *Reliability:* Not reported.  *Construct validity* of new questionnaire was demonstrated by small to moderate correlations with sleep diary and actigraphy total sleep time and time in bed.  Referred to previous literature validating actigraphy. | |
| 10 | Kaufmann *et al.* (2018) | Patients [bipolar disorder]; healthy controls | Diary;  Actigraphy on non-dominant wrist / continuous | Daily;  Not reported | 8 weeks (study 1)  3 weeks (study2) | Yes (sleep) | Yes | Compliance with sleep diary: 90%.  Compliance with actigraphy: | *Reliability:* The authors report that objective (actigraphy-derived) sleep interval midpoint values yielded reliable estimates comparable to self-reported chronotype.  *Validity:* Not reported. | |
| 11 | Krane-Gartiser *et al.* (2019) | Patients [bipolar disorder]; healthy controls | Diary;  Actigraphy / continuous | Daily;  1-min epochs | 21 days | Yes (sleep) | Yes | Not reported. | *Reliability:* Not reported.  *Validity:* Not reported. | |
| 12 | Langberg *et al.* (2019) | Patients [ADHD]; healthy controls | Diary^2^;  Actigraphy / continuous | Daily;  1-min epochs | 14 to 30 days | Yes (sleep) | Yes | Mean number of days for actigraphy: 14 days. Mean number of days for dairy: 15 days.  Actigraphy data: 8 participants did not have at least five schooldays and 15 participants did not have at least two weekend days.  Diary data: 12 participants did not have at least five schooldays and seven participants did not have at least two weekend days. | *Reliability:* Not reported.  *Validity:* Wear-time sensor built into the device was used in combination with a validation algorithm to maximize the accuracy of when the actigraph was physically being worn by finding the nonwear -times of based upon a threshold of consecutive zeros. | |
| 13 | Lovato *et al.* (2014) | Patients [insomnia] | Diary^2^;  Actigraphy on non-dominant wrist / continuous | Daily;  30-s epochs | 7 days  (5 times, i.e., screening, pretreatment, during treatment, post-treatment, follow-up) | Yes (sleep) | Yes | Not reported. | *Reliability:* Not reported.  *Validity:* Not reported. | |
| 14 | McMakin *et al.* (2019) | Patients [GAD; social anxiety disorder; separation anxiety disorder] | ESM;  Diary^2^;  Actigraphy / continuous | Not reported;  Daily;  Not reported; | 5 days  (5 times, i.e., baseline, 4 week, 8 week, 12 week, and post-treatment) | Yes (sleep) | Yes | *Compliance with diary*: 84% of diary sampling included five nights, 14% included four nights, 1% included three nights, and 1% included one night.  *Compliance with a*ctigraphy: The majority (66%) of actigraphy sampling included five nights, 18% included four nights, 7% included three nights, 5% included two nights, and 4% had one night. | *Reliability:* Not reported. Sleep diary and actigraphy analyses included the number of school nights during which data were collected as a covariate to account for differences in sleep patterns.  *Validity:* Not reported. | |
| 15 | McCrae *et al.* (2019) | Patients [fibromyalgia and insomnia] | Diary^2^;  Ambulatory polysomnography;  Actigraphy on non-dominant wrist / continuous | Daily to twice a day;  Not reported;  30-s epochs | 14 days  (3 times, i.e., baseline, post-treatment, and follow-up) | Yes (pain, sleep) | No | Not reported. | *Reliability:* Not reported.  *Validity:* Not reported. | |
| 16 | Merikangas *et al.* (2019) | Patients [bipolar I, bipolar II, MDD]; healthy controls | ESM with fixed sampling;  Actigraphy / continuous | 4 times / day;  1-min epoch | 14 days | Yes (mood, physical activity) | Yes | Not reported. | *Reliability:* Not reported.  *Validity:* Not reported. | |
| 17 | Owens *et al.* (2009) | Patients [ADHD]; healthy controls | Diary^2^;  Actigraphy at non-dominant wrist / continuous | Daily;  Not reported | 5 to 12 days | Yes (sleep) | Yes | Not reported. | *Reliability:* Not reported.  *Validity:* Not reported. | |
| 18 | Palmer *et al.* (2018) | Patients [GAD]; healthy controls | Diary^2^;  Actigraphy at wrist/ continuous | Daily;  Not reported | 7 days | Yes (sleep) | Yes | Compliance: 97 %.  Actigraphy data of participants with less than 5 days were excluded. | *Reliability:* Not reported.  *Validity:* Sleep diary used as a validation check for actigraphy data | |
| 19 | Prunas *et al.* (2019) | Patients [bipolar disorder] | Diary;  Actigraphy on non-dominant wrist / continuous | Daily;  1-min epochs | 21 days | Yes (sleep) | Yes | Not reported. | *Reliability:* Not reported.  *Validity:* No correlation was found of ADHD symptoms and actigraphy parameters. | |
| 20 | Robillard *et al.* (2014) | Patients [MDD; bipolar disorder] | Diary^2^;  Actigraphy / continuous | Daily;  30-s and 1-min epochs | 4 to 22 days | Yes (sleep) | Yes | Not reported. | *Reliability:* Not reported.  *Validity:* Not reported. | |
| 21 | Robillard *et al.* (2015) | Patients [anxiety disorder, MDD, bipolar disorder, psychotic disorder]; healthy controls | Diary^2^;  Actigraphy / continuous | Daily;  3-s and 1-min epochs | 4 to 22 days | Yes (sleep) | Yes | Not reported. | *Reliability:* Not reported.  *Validity:* Not reported. | |
| 22 | Robillard *et al.* (2016) | Patients [anxiety, MDD, bipolar disorder] | Diary^2^;  Actigraphy / continuous | Daily;  1-min epochs | 7 to 22 days | Yes (sleep) | Yes | Not reported. | *Reliability:* Not reported.  *Validity:* Not reported. | |
| 23 | Slyepchenko *et al.* (2019) | Patients [bipolar disorder; MDD] | Diary^2^;  Actigraphy at non-dominant wrist / continuous | Daily ;  1-min epochs | 15 days | Yes (sleep) | Yes | Not reported. | *Reliability:* Not reported.  *Validity:* Not reported. | |
| 24 | Soreca *et al.* (2016) | Patients [bipolar disorder] | Diary^2^;  Actigraphy on wrist / continuous | Daily;  Not reported | 7 days | Yes (sleep) | Yes | *Compliance for diary:* 88% of participants had at least diary data on 4 days.  *Compliance for actigraphy:* Not reported. | *Reliability:* Not reported.  *Validity:* Not reported. | |
| 25 | Titone *et al.* (2020) | High-risk population [bipolar disorder] | Diary;  Actigraphy on non-dominant wrist / continuous | Daily;  1-min epochs | 20 days | Yes (sleep, activity) | Yes | *Compliance*: 79%.  (Of 150 included participants, data of 118 was presented) | *Reliability:* Referred to the literature.  *Validity:* Referred to the literature that sleep parameters from actigraphy is moderately to strongly correlated with those derived from polysomnography. Actigraphy-measured sleep efficiency was positively correlated with self-reported sleep efficacy. | |
| 26 | Troxel *et al.* (2010) | Patients [insomnia]; healthy controls | Diary^2^;  Actigraphy at non-dominant wrist/ continuous | Daily;  1-min epochs | 14 days | Yes (sleep) | Yes | Not reported. | *Reliability:* Not reported.  *Validity:* Not reported. | |
| 27 | Verkooijen *et al.* (2017) | Patients [BPD]; healthy controls | Diary^2^;  Actigraphy at non-dominant wrist / continuous | Daily;  1-min epochs | 14 days | Yes (sleep) | Yes | Not reported. | *Reliability:* Not reported.  *Validity:* Not reported. | |
| 28 | Von Korff *et al.* (2012) | Patients [arthritis pain and insomnia] | Diary^2^;  Actigraphy at wirst / continuous | Daily;  1-min epochs | 7 days | Yes (sleep) | Yes | Response rate for post-intervention: 96.7%; for 9 month assessments: 92.9%. | *Reliability:* Not reported.  *Validity:* Not reported. | |
| 29 | Wallace *et al.* (2017) | Patients [GAD; social anxiety disorder; separation anxiety disorder] | ESM;  Diary^2^;  Actigraphy on non-dominant wrist / continuous | 2 (weekdays) to 4 (weekends)  Daily;  Not reported. | 5 days | Yes (PA,NA, sleep) | Yes | *Compliance with ESM*: 92%  1 (of 113) participants did not have any ESM data.  *Compliance with sleep diary*: 82% had data of 5 nights, 15% had data only for 1-4 nights and 4% had no sleep diary entries.  *Compliance with actigraphy:* 5 nights captured for 79% of participants; 1-4 nights for 16% and 0 nights for 5%. | *Reliability:* Referred to literature and used data of 5 nights only.  *Validity:* Referred to previous literature for validation of actigraphy. | |
| 30 | Wallen *et al.* (2019) | Patients [AUD] | Diary^2^;  Actigraphy at non-dominant wrist / continuous | Daily;  Not reported | 30 days | Yes (sleep) | Yes | *Compliance:* 84 % had six or more full nights of actigraphy recorded during the first seven days; 9% provided between one and five nights’ worth of data. 6% did not have any actigraphy data.  Only first 7 days used for analysis. | *Reliability:* Not reported.  *Validity:* Not reported. | |

*Note:* ^1^ Data aggregated refers to the calculation of a mean over the assessment period. ^2^ The study collected daily diary data over the course of actigraphy sampling, e.g., using paper diaries. However, the number of diary entries was too low in order to fulfill the criteria for a mixed study design (with at least 20 data points per modality). ADHD: Attention Deficit Hyper Disorder; AN: Anorexia nervosa; AUD: Alcohol Use Disorder; GAD: Generalized Anxiety Disorder; BN: bulimia nervosa; BPD: Borderline Personality Disorder; ESM: Experience Sampling Methodology; MDD: Major Depressive Disorder; SSD: Schizophrenia Spectrum Disorders; OCD: Obsessive Compulsive Disorder; EDNOS-AN: eating disorder not otherwise specified; mean compliance with diary method: 86%, mean compliance with actigraphy: 76%.

# **Table S5. Summary of studies from low and middle income countries (China, Brazil) with less than 100 participants.**

| **Number** | **Reference** | **Population** | **Data type** | **Sampling scheme** | **Prompts per day / sampling frequency** | **Assessment period** | **Primary outcome**  **is a time intensive measure** | **Data aggregated^1^** | **Measurement quality**  **(compliance, % data)** | **Psychometric properties (Reliability,**  **Validity)** |
| --- | --- | --- | --- | --- | --- | --- | --- | --- | --- | --- |
|  | **Sensor or mobile sensing** | | | | | | | | |  |
| 1 | Cheng et al., (2017) | Individuals at-risk from general population [suicidality, emotional distress] | ESM | Random |  | 12 months | Yes (affect) | Yes | Not reported. | *Reliability*: Not reported.  *Validity:* Not reported. |
| 2 | Yin *et al.* (2017) | Patients [insomnia] | Actigraphy | continuous |  | 4 weeks | Yes (sleep) | Yes | Not reported. | *Reliability:* Not reported.  *Validity:* Not reported. |
|  | **Combination of active and passive methods** | | | | | | | | |  |
| 3 | Chung et al., (2018) | Patients [insomnia] | Diary^2^; Actigraphy | time-contingent; continuous | Daily  Actigraphy: 1-min epochs | 6 days | Yes (sleep) | Yes | Dropout rate: 8.9%. | *Reliability:* Not reported.  *Validity:* Not reported. |
| 4 | D’Aurea et al., (2019) | Individuals at-risk from general population [insomnia] | Diary^2^; Actigraphy | time-contingent; continuous | Actigraphy: 1-min epochs | 15 days | Yes (sleep) | Yes | Not reported. | *Reliability:* Not reported.  *Validity:* Not reported. |
| 5 | Yueng et al., (2011) | Patients [MDD, insomnia] | Diary^2^; Actigraphy | time-contingent; continuous | Actigraphy: 1-min epochs | 6 days  Actigraphy: 3 days | Yes (sleep) | Yes | Drop-out rate: 9%; 3.8% withdrew from study at 4 week follow-up. | *Reliability:* Actigraphy: Not reported, scales: very good.  *Validity*: Not reported. |

*Note:* ^1^Data aggregated refers to the calculation of a mean over the assessment period. ^2^The study collected daily diary data over the course of actigraphy sampling. However, the number of diary entries was too low in order to fulfill the criteria for a mixed study design (with at least 20 data points per modality). ESM: Experience Sampling Methodology; MDD: Major Depressive Disorder.

# **References**

**Aikens, J. E., Trivedi, R., Heapy, A., Pfeiffer, P. N. & Piette, J. D.** (2015). Potential Impact of Incorporating a Patient-Selected Support Person into mHealth for Depression. *Journal of General Internal Medicine* **30**, 797-803. doi: [10.1007/s11606-015-3208-7](https://doi.org/10.1007/s11606-015-3208-7)

**Andrewes, H. E., Hulbert, C., Cotton, S. M., Betts, J. & Chanen, A. M.** (2017). An ecological momentary assessment investigation of complex and conflicting emotions in youth with borderline personality disorder. *Psychiatry Research* **252**, 102-110. doi: [10.1016/j.psychres.2017.01.100](https://doi.org/10.1016/j.psychres.2017.01.100)

**Anestis, M. D., Selby, E. A., Crosby, R. D., Wonderlich, S. A., Engel, S. G. & Joiner, T. E.** (2010). A comparison of retrospective self-report versus ecological momentary assessment measures of affective lability in the examination of its relationship with bulimic symptomatology. *Behavior Research and Therapy* **48**, 607-13. doi: [10.1016/j.brat.2010.03.012](https://doi.org/10.1016/j.brat.2010.03.012)

**Baerg, S., Cairney, J., Hay, J., Rempel, L., Mahlberg, N. & Faught, B. E.** (2011). Evaluating physical activity using accelerometry in children at risk of developmental coordination disorder in the presence of attention deficit hyperactivity disorder. *Research in Developmental Disabilities* **32**, 1343-50. doi: [10.1016/j.ridd.2011.02.009](https://doi.org/10.1016/j.ridd.2011.02.009)

**Banihashemi, N., Robillard, R., Yang, J., Carpenter, J. S., Hermens, D. F., Naismith, S. L., … & Hickie, I. B.** (2016). Quantifying the effect of body mass index, age, and depression severity on 24-h activity patterns in persons with a lifetime history of affective disorders. *BMC Psychiatry* **16**, 317. doi: [10.1186/s12888-016-1023-2](https://bmcpsychiatry.biomedcentral.com/articles/10.1186/s12888-016-1023-2)

**Becker, K. R., Fischer, S., Crosby, R. D., Engel, S. G. & Wonderlich, S. A.** (2018). Dimensional analysis of emotion trajectories before and after disordered eating behaviors in a sample of women with bulimia nervosa. *Psychiatry Research* **268**, 490-500. doi: [10.1016/j.psychres.2018.08.008](https://doi.org/10.1016/j.psychres.2018.08.008)

**Becker, S. P., Langberg, J. M., Eadeh, H. M., Isaacson, P. A. & Bourchtein, E.** (2019). Sleep and daytime sleepiness in adolescents with and without ADHD: differences across ratings, daily diary, and actigraphy. *Journal of Child Psychology and Psychiatry* **60**, 1021-1031. doi: [10.1111/jcpp.13061](https://doi.org/10.1111/jcpp.13061)

**Benard, V., Etain, B., Vaiva, G., Boudebesse, C., Yeim, S., Benizri, C., … & Geoffroy, P. A.** (2019). Sleep and circadian rhythms as possible trait markers of suicide attempt in bipolar disorders: An actigraphy study. *Journal of Affective Disorders* **244**, 1-8. doi: [10.1016/j.jad.2018.09.054](https://doi.org/10.1016/j.jad.2018.09.054)

**Berg, K. C., Cao, L., Crosby, R. D., Engel, S. G., Peterson, C. B., Crow, S. J., … & Wonderlich, S. A.** (2017). Negative affect and binge eating: Reconciling differences between two analytic approaches in ecological momentary assessment research. *International Journal of Eating Disorders* **50**, 1222-1230. doi: [10.1002/eat.22770](https://psycnet.apa.org/doi/10.1002/eat.22770)

**Berg, K. C., Crosby, R. D., Cao, L., Peterson, C. B., Engel, S. G., Mitchell, J. E. & Wonderlich, S. A.** (2013). Facets of negative affect prior to and following binge-only, purge-only, and binge/purge events in women with bulimia nervosa. *Journal of Abnormal Psychology* **122**, 111-8. doi: [10.1037/a0029703](https://psycnet.apa.org/doi/10.1037/a0029703)

**Bergwerff, C. E., Luman, M. & Oosterlaan, J.** (2016). No objectively measured sleep disturbances in children with attention-deficit/hyperactivity disorder. *Journal of Sleep Research* **25**, 534-540. doi: [**10.1111/jsr.12399**](https://doi.org/10.1111/jsr.12399)

**Berner, L. A., Crosby, R. D., Cao, L., Engel, S. G., Lavender, J. M., Mitchell, J. E. & Wonderlich, S. A.** (2017). Temporal associations between affective instability and dysregulated eating behavior in bulimia nervosa. *Journal of Psychiatric Research* **92**, 183-190. doi: [10.1016/j.jpsychires.2017.04.009](https://doi.org/10.1016/j.jpsychires.2017.04.009)

**Bilderbeck, A. C., Atkinson, L. Z., McMahon, H. C., Voysey, M., Simon, J., Price, J., … & Goodwin, G. M.** (2016). Psychoeducation and online mood tracking for patients with bipolar disorder: A randomised controlled trial. *Journal of Affective Disorders* **205**, 245-251. doi: [10.1016/j.jad.2016.06.064](https://doi.org/10.1016/j.jad.2016.06.064)

**Birnbaum, M. L., Ernala, S. K., Rizvi, A. F., De Choudhury, M. & Kane, J. M.** (2017). A Collaborative Approach to Identifying Social Media Markers of Schizophrenia by Employing Machine Learning and Clinical Appraisals. *Journal of Medical Internet Research* **19**, e289. [doi:10.2196/jmir.7956](https://doi.org/10.2196/jmir.7956)

**Blake, M., Waloszek, J. M., Schwartz, O., Raniti, M., Simmons, J. G., Blake, … & Allen, N. B.** (2016). The SENSE study: Post intervention effects of a randomized controlled trial of a cognitive-behavioral and mindfulness-based group sleep improvement intervention among at-risk adolescents. *Journal of Consulting and Clinical Psychology* **84**, 1039-1051. doi: [10.1037/ccp0000142](https://psycnet.apa.org/doi/10.1037/ccp0000142)

**Blake, M. J., Blake, L. M., Schwartz, O., Raniti, M., Waloszek, J. M., Murray, G., … & Allen, N. B.** (2018). Who benefits from adolescent sleep interventions? Moderators of treatment efficacy in a randomized controlled trial of a cognitive-behavioral and mindfulness-based group sleep intervention for at-risk adolescents. *Journal of Child Psychology and Psychiatry* **59**, 637-649. doi: [**10.1111/jcpp.12842**](https://doi.org/10.1111/jcpp.12842)

**Blake, M. J., Snoep, L., Raniti, M., Schwartz, O., Waloszek, J. M., Simmons, J. G., … & Allen, N. B.** (2017). A cognitive-behavioral and mindfulness-based group sleep intervention improves behavior problems in at-risk adolescents by improving perceived sleep quality. *Behavior Research and Therapy* **99**, 147-156. doi: [10.1016/j.brat.2017.10.006](https://doi.org/10.1016/j.brat.2017.10.006)

**Block, V. J., Meyer, A. H., Miche, M., Mikoteit, T., Hoyer, J., Imboden, C., … & Gloster, A. T.** (2020). The effect of anticipatory stress and openness and engagement on subsequently perceived sleep quality-An Experience Sampling Method study. *Journal of Sleep Research* **29**, e12957. doi: [10.1111/jsr.12957](https://psycnet.apa.org/doi/10.1111/jsr.12957)

**Blum, L. H., Vakhrusheva, J., Saperstein, A., Khan, S., Chang, R. W., Hansen, M. C., … & Kimhy, D.** (2015). Depressed mood in individuals with schizophrenia: A comparison of retrospective and real-time measures. *Psychiatry Research* **227**, 318-23. doi: [10.1016/j.psychres.2015.03.008](https://doi.org/10.1016/j.psychres.2015.03.008)

**Blytt, K. M., Bjorvatn, B., Husebo, B. & Flo, E.** (2018). Effects of pain treatment on sleep in nursing home patients with dementia and depression: A multicenter placebo-controlled randomized clinical trial. *International Journal of Geriatric Psychiatry* **33**, 663-670. doi: [10.1002/gps.4839](https://doi.org/10.1002/gps.4839)

**Bracht, T., Heidemeyer, K., Koschorke, P., Horn, H., Razavi, N., Wopfner, A., … & Walther, S.** (2012). Comparison of objectively measured motor behavior with ratings of the motor behavior domain of the Bern Psychopathology Scale (BPS) in schizophrenia. *Psychiatry Research* **198**, 224-9. doi: [10.1016/j.psychres.2011.12.038](https://doi.org/10.1016/j.psychres.2011.12.038)

**Bratland-Sanda, S., Martinsen, E. W., Rosenvinge, J. H., Ro, O., Hoffart, A. & Sundgot-Borgen, J.** (2011). Exercise dependence score in patients with longstanding eating disorders and controls: the importance of affect regulation and physical activity intensity. *European Eating Disorders Review* **19**, 249-55. doi: [10.1002/erv.971](https://doi.org/10.1002/erv.971)

**Chapman, A. L., Rosenthal, M. Z., Dixon-Gordon, K. L., Turner, B. J. & Kuppens, P.** (2017). Borderline Personality Disorder and the Effects of Instructed Emotional Avoidance or Acceptance in Daily Life. *Journal of Personality Disorders* **31**, 483-502. Retrieved from: [Chapman2017BPDAT.pdf (kuleuven.be)](https://ppw.kuleuven.be/okp/_pdf/Chapman2017BPDAT.pdf)

**Chen, L. J., Steptoe, A., Chung, M. S. & Ku, P. W.** (2016). Association between actigraphy-derived physical activity and cognitive performance in patients with schizophrenia. *Psychological Medicine* **46**, 2375-84. doi: [10.1017/S0033291716000921](https://doi.org/10.1017/s0033291716000921)

**Cheng, Q., Li, T. M., Kwok, C. L., Zhu, T. & Yip, P. S.** (2017). Assessing Suicide Risk and Emotional Distress in Chinese Social Media: A Text Mining and Machine Learning Study. *Journal of Medical Internet Research* **19**, e243. doi: [10.2196/jmir.7276](https://doi.org/10.2196/jmir.7276)

**Choi, K. W., Chen, C. Y., Stein, M. B., Klimentidis, Y. C., Wang, M. J., Koenen, K. C., … & Major Depressive Disorder Working Group of the Psychiatric Genomics, C.** (2019). Assessment of Bidirectional Relationships Between Physical Activity and Depression Among Adults: A 2-Sample Mendelian Randomization Study. *JAMA Psychiatry* **76**, 399-408. doi: [10.1001/jamapsychiatry.2018.4175](https://doi.org/10.1001/jamapsychiatry.2018.4175)

**Chung, K. F., Yeung, W. F., Yu, B. Y., Leung, F. C., Zhang, S. P., Zhang, Z. J., … & Yiu, G. C.** (2018). Acupuncture with or without combined auricular acupuncture for insomnia: a randomised, waitlist-controlled trial. *Acupuncture in Medicine* **36**, 2-13. doi: [10.1136/acupmed-2017-011371](https://doi.org/10.1136/acupmed-2017-011371)

**Chung, K. F., Yeung, W. F., Yu, Y. M., Yung, K. P., Zhang, S. P., Zhang, Z. J., … & Chan, L. W.** (2015). Acupuncture for residual insomnia associated with major depressive disorder: a placebo- and sham-controlled, subject- and assessor-blind, randomized trial. *Journal of Clinical Psychiatry* **76**, e752-60. doi; [10.4088/JCP.14m09124](https://doi.org/10.4088/jcp.14m09124)

**Collip, D., Nicolson, N. A., Lardinois, M., Lataster, T., van Os, J., Myin-Germeys, I. & G.R.O.U.P** (2011a). Daily cortisol, stress reactivity and psychotic experiences in individuals at above average genetic risk for psychosis. *Psychological Medicine* **41**, 2305-15. doi:10.1017/S0033291711000602

**Collip, D., Oorschot, M., Thewissen, V., Van Os, J., Bentall, R. & Myin-Germeys, I.** (2011b). Social world interactions: how company connects to paranoia. *Psychological Medicine* **41**, 911-21. doi:10.1017/S0033291710001558

**Collip, D., van Winkel, R., Peerbooms, O., Lataster, T., Thewissen, V., Lardinois, M., … & Myin-Germeys, I.** (2011c). COMT Val158Met-stress interaction in psychosis: role of background psychosis risk. *CNS Neuroscience and Therapeutics* **17**, 612-9. doi: [10.1111/j.1755-5949.2010.00213.x](https://doi.org/10.1111/j.1755-5949.2010.00213.x)

**Collip, D., Wigman, J. T., van Os, J., Oorschot, M., Jacobs, N., Derom, C., … & Myin-Germeys, I.** (2014). Positive emotions from social company in women with persisting subclinical psychosis: lessons from daily life. *Acta Psychiatrica Scandinavica* **129**, 202-10. doi: [10.1111/acps.12151](https://doi.org/10.1111/acps.12151)

**Crosby, R. D., Wonderlich, S. A., Engel, S. G., Simonich, H., Smyth, J. & Mitchell, J. E.** (2009). Daily mood patterns and bulimic behaviors in the natural environment. *Behavior Research and Therapy* **47**, 181-8. doi: [10.1016/j.brat.2008.11.006](https://doi.org/10.1016/j.brat.2008.11.006)

**D'Aurea, C. V. R., Poyares, D., Passos, G. S., Santana, M. G., Youngstedt, S. D., Souza, A. A., … & de Mello, M. T.** (2019). Effects of resistance exercise training and stretching on chronic insomnia. *Brazilian Journal of Psychiatry* **41**, 51-57. doi: [10.1590/1516-4446-2018-0030](https://doi.org/10.1590/1516-4446-2018-0030)

**de Bruin, E. J., Bogels, S. M., Oort, F. J. & Meijer, A. M.** (2018). Improvements of adolescent psychopathology after insomnia treatment: results from a randomized controlled trial over 1 year. *Journal of Child Psychology and Psychiatry* **59**, 509-522. doi: [10.1111/jcpp.12834](https://doi.org/10.1111/jcpp.12834)

**De Young, K. P., Lavender, J. M., Crosby, R. D., Wonderlich, S. A., Engel, S. G., Mitchell, J. E., …& Le Grange, D.** (2014). Bidirectional associations between binge eating and restriction in anorexia nervosa. An ecological momentary assessment study. *Appetite* **83**, 69-74. doi: [10.1016/j.appet.2014.08.014](https://psycnet.apa.org/doi/10.1016/j.appet.2014.08.014)

**De Young, K. P., Lavender, J. M., Steffen, K., Wonderlich, S. A., Engel, S. G., Mitchell, J. E., … & Crosby, R. D.** (2013a). Restrictive eating behaviors are a nonweight-based marker of severity in anorexia nervosa. *International Journal of Eating Disorders* **46**, 849-54. doi: [10.1002/eat.22163](https://doi.org/10.1002%2Feat.22163)

**De Young, K. P., Lavender, J. M., Wonderlich, S. A., Crosby, R. D., Engel, S. G., Mitchell, J. E., … & Le Grange, D.** (2013b). Moderators of post-binge eating negative emotion in eating disorders. *Journal of Psychiatric Research* **47**, 323-8. doi: [10.1016/j.jpsychires.2012.11.012](https://doi.org/10.1016%2Fj.jpsychires.2012.11.012)

**Difrancesco, S., Lamers, F., Riese, H., Merikangas, K. R., Beekman, A. T. F., van Hemert, A. M., … & Penninx, B.** (2019). Sleep, circadian rhythm, and physical activity patterns in depressive and anxiety disorders: A 2-week ambulatory assessment study. *Depression and Anxiety* **36**, 975-986. doi: [10.1002/da.22949](https://doi.org/10.1002/da.22949)

**Duffy, A., Keown-Stoneman, C. D., Goodday, S. M., Saunders, K., Horrocks, J., Grof, P., … & Geddes, J.** (2019). Daily and weekly mood ratings using a remote capture method in high-risk offspring of bipolar parents: Compliance and symptom monitoring. *Bipolar Disorder* **21**, 159-167. doi: [10.1111/bdi.12721](https://doi.org/10.1111/bdi.12721)

**Duncan, M. J., Arbour-Nicitopoulos, K., Subramanieapillai, M., Remington, G. & Faulkner, G.** (2017). Revisiting the International Physical Activity Questionnaire (IPAQ): Assessing physical activity among individuals with schizophrenia. *Schizophrenia Research* **179**, 2-7. doi: [10.1016/j.schres.2016.09.010](https://doi.org/10.1016/j.schres.2016.09.010)

**Eichstaedt, J. C., Smith, R. J., Merchant, R. M., Ungar, L. H., Crutchley, P., Preotiuc-Pietro, D., … & Schwartz, H. A.** (2018). Facebook language predicts depression in medical records. *Proceedings of the National Academy of Science U S A* **115**, 11203-11208. doi: [10.1073/pnas.1802331115](https://doi.org/10.1073/pnas.1802331115)

**Engel, S. G., Boseck, J. J., Crosby, R. D., Wonderlich, S. A., Mitchell, J. E., Smyth, J., … & Steiger, H.** (2007). The relationship of momentary anger and impulsivity to bulimic behavior. *Behavior Research and Therapy* **45**, 437-47. doi: [10.1016/j.brat.2006.03.014](https://psycnet.apa.org/doi/10.1016/j.brat.2006.03.014)

**Engel, S. G., Wonderlich, S. A., Crosby, R. D., Mitchell, J. E., Crow, S., Peterson, C. B., … & Gordon, K. H.** (2013). The role of affect in the maintenance of anorexia nervosa: evidence from a naturalistic assessment of momentary behaviors and emotion. *Journal of Abnormal Psychology* **122**, 709-19. doi: [10.1037/a0034010](https://psycnet.apa.org/doi/10.1037/a0034010)

**Erwin, M. C., Dennis, P. A., Coughlin, L. N., Calhoun, P. S. & Beckham, J. C.** (2019). Examining the relationship between negative affect and posttraumatic stress disorder symptoms among smokers using ecological momentary assessment. *Journal of Affective Disorders* **253**, 285-291. doi: [10.1016/j.jad.2019.04.035](https://doi.org/10.1016/j.jad.2019.04.035)

**Faedda, G. L., Ohashi, K., Hernandez, M., McGreenery, C. E., Grant, M. C., Baroni, A., ... & Teicher, M. H.** (2016). Actigraph measures discriminate pediatric bipolar disorder from attention-deficit/hyperactivity disorder and typically developing controls. *Journal of Child Psychology and Psychiatry* **57**, 706-16. doi: [10.1111/jcpp.12520](https://doi.org/10.1111%2Fjcpp.12520)

**Fang, S. H., Suzuki, K., Lim, C. L., Chung, M. S., Ku, P. W. & Chen, L. J.** (2016). Associations between sleep quality and inflammatory markers in patients with schizophrenia. *Psychiatry Research* **246**, 154-160. doi: [10.1016/j.psychres.2016.09.032](https://doi.org/10.1016/j.psychres.2016.09.032)

**Fatseas, M., Serre, F., Swendsen, J. & Auriacombe, M.** (2018). Effects of anxiety and mood disorders on craving and substance use among patients with substance use disorder: An ecological momentary assessment study. *Drug and Alcohol Dependence* **187**, 242-248, doi: [10.1016/j.drugalcdep.2018.03.008](https://doi.org/10.1016/j.drugalcdep.2018.03.008)

**Fitzsimmons-Craft, E. E., Accurso, E. C., Ciao, A. C., Crosby, R. D., Cao, L., Pisetsky, E. M., … & Wonderlich, S. A.** (2015). Restrictive eating in anorexia nervosa: Examining maintenance and consequences in the natural environment. *International Journal of Eating Disorders* **48**, 923-31. doi: [10.1002/eat.22439](https://psycnet.apa.org/doi/10.1002/eat.22439)

**Fitzsimmons-Craft, E. E., Firebaugh, M. L., Graham, A. K., Eichen, D. M., Monterubio, G. E., Balantekin, K. N., … & Wilfley, D. E.** (2019). State-wide university implementation of an online platform for eating disorders screening and intervention. *Psychological Services* **16**, 239-249. doi: [10.1037/ser0000264](https://doi.org/10.1037/ser0000264)

**Friedmann, F., Santangelo, P., Ebner-Priemer, U., Hill, H., Neubauer, A. B., Rausch, S., … & Priebe, K.** (2020). Life within a limited radius: Investigating activity space in women with a history of child abuse using global positioning system tracking. *PLoS One* **15**, e0232666. doi: [10.1371/journal.pone.0232666](https://psycnet.apa.org/doi/10.1371/journal.pone.0232666)

**Frissen, A., Lieverse, R., Drukker, M., Delespaul, P., Lataster, T., Myin-Germeys, I. & van Os, J.** (2014). Evidence that childhood urban environment is associated with blunted stress reactivity across groups of patients with psychosis, relatives of patients and controls. *Social Psychiatry and Psychiatric Epidemiology* **49**, 1579-87. doi: 10.1007/s00127-014-0859-3

**Geoffroy, P. A., Micoulaud Franchi, J. A., Maruani, J., Philip, P., Boudebesse, C., Benizri, C., … & Etain, B.** (2019). Clinical characteristics of obstructive sleep apnea in bipolar disorders. *Journal of Affective Disorders* **245**, 1-7. doi: 10.1016/j.jad.2018.10.096

**George, M. J., Russell, M. A., Piontak, J. R. & Odgers, C. L.** (2018). Concurrent and Subsequent Associations Between Daily Digital Technology Use and High-Risk Adolescents' Mental Health Symptoms. *Child Development* **89**, 78-88. doi: [10.1111/cdev.12819](https://doi.org/10.1111/cdev.12819)

**Gerritsen, C., Bagby, R. M., Sanches, M., Kiang, M., Maheandiran, M., Prce, I. & Mizrahi, R.** (2019). Stress precedes negative symptom exacerbations in clinical high risk and early psychosis: A time-lagged experience sampling study. *Schizophrenia Research* **210**, 52-58. doi: 10.1016/j.schres.2019.06.015

**Geschwind, N., Peeters, F., Drukker, M., van Os, J. & Wichers, M.** (2011). Mindfulness training increases momentary positive emotions and reward experience in adults vulnerable to depression: a randomized controlled trial. *Journal of Consulting and Clinical Psychology* **79**, 618-28. doi: [10.1037/a0024595](https://doi.org/10.1037/a0024595)

**Glaser, J. P., Van Os, J., Mengelers, R. & Myin-Germeys, I.** (2008). A momentary assessment study of the reputed emotional phenotype associated with borderline personality disorder. *Psychological Medicine* **38**, 1231-9. doi: 10.1017/S0033291707002322

**Glaser, J. P., Van Os, J., Thewissen, V. & Myin-Germeys, I.** (2010). Psychotic reactivity in borderline personality disorder. *Acta Psychiatrica Scandinavica* **121**, 125-34. doi: 10.1111/j.1600-0447.2009.01427.x

**Goldschmidt, A. B., Accurso, E. C., Schreiber-Gregory, D. N., Crosby, R. D., Cao, L., Engel, S. G., … & Wonderlich, S. A.** (2015). Behavioral, emotional, and situational context of purging episodes in anorexia nervosa. *International Journal of Eating Disorders* **48**, 341-4. doi: [https://doi.org/10.1002/eat.22381](https://psycnet.apa.org/doi/10.1002/eat.22381)

**Goldschmidt, A. B., Peterson, C. B., Wonderlich, S. A., Crosby, R. D., Engel, S. G., Mitchell, J. E., … & Berg, K. C.** (2013). Trait-level and momentary correlates of bulimia nervosa with a history of anorexia nervosa. *International Journal of Eating Disorders* **46**, 140-6. doi: [10.1002/eat.22054](https://doi.org/10.1002/eat.22054)

**Goldschmidt, A. B., Wonderlich, S. A., Crosby, R. D., Cao, L., Engel, S. G., Lavender, J. M., … & Le Grange, D.** (2014a). Latent profile analysis of eating episodes in anorexia nervosa. *Journal of Psychiatric Research* **53**, 193-9. doi: [10.1016/j.jpsychires.2014.02.019](https://doi.org/10.1016%2Fj.jpsychires.2014.02.019)

**Goldschmidt, A. B., Wonderlich, S. A., Crosby, R. D., Engel, S. G., Lavender, J. M., Peterson, C. B., … & Mitchell, J. E.** (2014b). Ecological momentary assessment of stressful events and negative affect in bulimia nervosa. *Journal of Consulting and Clinical Psychology* **82**, 30-9. doi:  [10.1037/a0034974](https://doi.org/10.1037%2Fa0034974)

**Goodlin-Jones, B. L., Waters, S. & Anders, T. F.** (2009). Objective sleep measurement in typically and atypically developing preschool children with ADHD-like profiles. *Child Psychiatry and Human Development* **40**, 257-68. doi: [10.1007/s10578-009-0124-2](https://doi.org/10.1007/s10578-009-0124-2)

**Granholm, E., Ben-Zeev, D., Fulford, D. & Swendsen, J.** (2013). Ecological Momentary Assessment of social functioning in schizophrenia: impact of performance appraisals and affect on social interactions. *Schizophrenia Research* **145**, 120-4. doi: [10.1016/j.schres.2013.01.005](https://doi.org/10.1016/j.schres.2013.01.005)

**Gregory, A. M., Cousins, J. C., Forbes, E. E., Trubnick, L., Ryan, N. D., Axelson, D. A., … & Dahl, R. E.** (2011). Sleep items in the child behavior checklist: a comparison with sleep diaries, actigraphy, and polysomnography. *Journal of the American Academy of Child and Adolescent Psychiatry* **50**, 499-507. doi: [10.1016/j.jaac.2011.02.003](https://doi.org/10.1016/j.jaac.2011.02.003)

**Hartmann, J. A., Wichers, M., Menne-Lothmann, C., Kramer, I., Viechtbauer, W., Peeters, F., … & Simons, C. J.** (2015). Experience sampling-based personalized feedback and positive affect: a randomized controlled trial in depressed patients. *PLoS One* **10**, e0128095. doi: [10.1371/journal.pone.0128095](https://doi.org/10.1371/journal.pone.0128095)

**Haynos, A. F., Berg, K. C., Cao, L., Crosby, R. D., Lavender, J. M., Utzinger, L. M., ... & Crow, S. J.** (2017). Trajectories of higher- and lower-order dimensions of negative and positive affect relative to restrictive eating in anorexia nervosa. *Journal of Abnormal Psychology* **126**, 495-505. doi: [10.1037/abn0000202](https://doi.org/10.1037%2Fabn0000202)

**Haynos, A. F., Crosby, R. D., Engel, S. G., Lavender, J. M., Wonderlich, S. A., Mitchell, J. E., … Le Grange, D.** (2015). Initial test of an emotional avoidance model of restriction in anorexia nervosa using ecological momentary assessment. *Journal of Psychiatric Research* **68**, 134-9. doi: [10.1016/j.jpsychires.2015.06.016](https://doi.org/10.1016/j.jpsychires.2015.06.016)

**Heininga, V. E., van Roekel, E., Ahles, J. J., Oldehinkel, A. J. & Mezulis, A. H.** (2017). Positive affective functioning in anhedonic individuals' daily life: Anything but flat and blunted. *Journal of Affective Disorders* **218**, 437-445. doi: [10.1016/j.jad.2017.04.029](https://doi.org/10.1016/j.jad.2017.04.029)

**Hensch, T., Wozniak, D., Spada, J., Sander, C., Ulke, C., Wittekind, D. A., … & Hegerl, U.** (2019). Vulnerability to bipolar disorder is linked to sleep and sleepiness. *Translational Psychiatry* **9**, 294. doi: [10.1038/s41398-019-0632-1](https://doi.org/10.1038/s41398-019-0632-1)

**Hswen, Y., Naslund, J. A., Brownstein, J. S. & Hawkins, J. B.** (2018). Online Communication about Depression and Anxiety among Twitter Users with Schizophrenia: Preliminary Findings to Inform a Digital Phenotype Using Social Media. *Psychiatry Quaterly* **89**, 569-580. doi: [10.1007/s11126-017-9559-y](https://doi.org/10.1007%2Fs11126-017-9559-y)

**Hswen, Y., Naslund, J. A., Chandrashekar, P., Siegel, R., Brownstein, J. S. & Hawkins, J. B.** (2017). Exploring online communication about cigarette smoking among Twitter users who self-identify as having schizophrenia. *Psychiatry Research* **257**, 479-484. doi: [10.1016/j.psychres.2017.08.002](https://doi.org/10.1016%2Fj.psychres.2017.08.002)

**Hvolby, A., Jorgensen, J. & Bilenberg, N.** (2008). Actigraphic and parental reports of sleep difficulties in children with attention-deficit/hyperactivity disorder. *Archives of Pediatric and Adolescent Medicine* **162**, 323-9. doi: [10.1001/archpedi.162.4.323](https://doi.org/10.1001/archpedi.162.4.323)

**Jahng, S., Solhan, M. B., Tomko, R. L., Wood, P. K., Piasecki, T. M. & Trull, T. J.** (2011). Affect and alcohol use: an ecological momentary assessment study of outpatients with borderline personality disorder. *Journal of Abnormal Psychology* **120**, 572-84. doi: [10.1037/a0024686](https://doi.org/10.1037%2Fa0024686)

**Janssens, M., Lataster, T., Simons, C. J., Oorschot, M., Lardinois, M., van Os, J., … & Group** (2012). Emotion recognition in psychosis: no evidence for an association with real world social functioning. *Schizophrenia Research* **142**, 116-21. doi: [10.1016/j.schres.2012.10.003](https://doi.org/10.1016/j.schres.2012.10.003)

**Jappe, L. M., Cao, L., Crosby, R. D., Crow, S. J., Peterson, C. B., Le Grange, D., … & Wonderlich, S. A.** (2014). Stress and eating disorder behavior in anorexia nervosa as a function of menstrual cycle status. *International Journal of Eating Disorders* **47**, 181-8. doi: [10.1002/eat.22211](https://doi.org/10.1002/eat.22211)

**Johns, J. T., Di, J., Merikangas, K., Cui, L., Swendsen, J. & Zipunnikov, V.** (2019). Fragmentation as a novel measure of stability in normalized trajectories of mood and attention measured by ecological momentary assessment. *Psychological Assessment* **31**, 329-339. doi: [10.1037/pas0000661](https://psycnet.apa.org/doi/10.1037/pas0000661)

**Kaplan, K. A., Plante, D. T., Cook, J. D. & Harvey, A. G.** (2019). Development and validation of the Hypersomnia Severity Index (HSI): A measure to assess hypersomnia severity and impairment in psychiatric disorders. *Psychiatry Research* **281**, 112547. doi: [10.1016/j.psychres.2019.112547](https://doi.org/10.1016/j.psychres.2019.112547)

**Karr, T. M., Crosby, R. D., Cao, L., Engel, S. G., Mitchell, J. E., Simonich, H. & Wonderlich, S. A.** (2013). Posttraumatic stress disorder as a moderator of the association between negative affect and bulimic symptoms: an ecological momentary assessment study. *Comprehensive Psychiatry* **54**, 61-9. doi: [10.1016/j.comppsych.2012.05.011](https://psycnet.apa.org/doi/10.1016/j.comppsych.2012.05.011)

**Kaufmann, C. N., Gershon, A., Depp, C. A., Miller, S., Zeitzer, J. M. & Ketter, T. A.** (2018). Daytime midpoint as a digital biomarker for chronotype in bipolar disorder. *Journal of Affective Disorders* **241**, 586-591. doi: [10.1016/j.jad.2018.08.032](https://doi.org/10.1016%2Fj.jad.2018.08.032)

**Keyes, A., Woerwag-Mehta, S., Bartholdy, S., Koskina, A., Middleton, B., Connan, F., … & Campbell, I. C.** (2015). Physical activity and the drive to exercise in anorexia nervosa. *International Journal of Eating Disorders* **48**, 46-54. doi: [10.1002/eat.22354](https://doi.org/10.1002/eat.22354)

**Khazanov, G. K., Ruscio, A. M. & Swendsen, J.** (2019). The "Brightening" Effect: Reactions to Positive Events in the Daily Lives of Individuals With Major Depressive Disorder and Generalized Anxiety Disorder. *Behavior Therapy* **50**, 270-284. doi: [10.1016/j.beth.2018.05.008](https://psycnet.apa.org/doi/10.1016/j.beth.2018.05.008)

**Kimhy, D., Vakhrusheva, J., Khan, S., Chang, R. W., Hansen, M. C., Ballon, J. S., … & Gross, J. J.** (2014). Emotional granularity and social functioning in individuals with schizophrenia: an experience sampling study. *Journal of Psychiatric Research* **53**, 141-8. doi: [10.1016/j.jpsychires.2014.01.020](https://doi.org/10.1016/j.jpsychires.2014.01.020)

**Klippel, A., Myin-Germeys, I., Chavez-Baldini, U., Preacher, K. J., Kempton, M., Valmaggia, L., … & Reininghaus, U.** (2017). Modeling the Interplay Between Psychological Processes and Adverse, Stressful Contexts and Experiences in Pathways to Psychosis: An Experience Sampling Study. *Schizophrenia Bulletin* **43**, 302-315. doi: [10.1093/schbul/sbw185](https://doi.org/10.1093%2Fschbul%2Fsbw185)

**Klippel, A., Viechtbauer, W., Reininghaus, U., Wigman, J., van Borkulo, C., Merge, Myin-Germeys, I. & Wichers, M.** (2018). The Cascade of Stress: A Network Approach to Explore Differential Dynamics in Populations Varying in Risk for Psychosis. *Schizophrenia Bulletin* **44**, 328-337. doi: [10.1093/schbul/sbx037](https://doi.org/10.1093/schbul/sbx037)

**Kordy, H., Wolf, M., Aulich, K., Burgy, M., Hegerl, U., Husing, J., … & Backenstrass, M.** (2016). Internet-Delivered Disease Management for Recurrent Depression: A Multicenter Randomized Controlled Trial. *Psychotherapy and Psychosomatcs* **85**, 91-8. doi: [10.1159/000441951](https://doi.org/10.1159/000441951)

**Krane-Gartiser, K., Scott, J., Nevoret, C., Benard, V., Benizri, C., Brochard, H., … & Etain, B.** (2019). Which actigraphic variables optimally characterize the sleep-wake cycle of individuals with bipolar disorders? *Acta Psychiatrica Scandinavica* **139**, 269-279. doi: [10.1111/acps.13003](https://doi.org/10.1111/acps.13003)

**Kuepper, R., Oorschot, M., Myin-Germeys, I., Smits, M., van Os, J. & Henquet, C.** (2013). Is psychotic disorder associated with increased levels of craving for cannabis? An Experience Sampling study. *Acta Psychiatrica Scandinavica* **128**, 448-56. doi: [10.1111/acps.12078](https://doi.org/10.1111/acps.12078)

**Langberg, J. M., Breaux, R. P., Cusick, C. N., Green, C. D., Smith, Z. R., Molitor, S. J. & Becker, S. P.** (2019). Intraindividual variability of sleep/wake patterns in adolescents with and without attention-deficit/hyperactivity disorder. *Journal of Child Psychology and Psychiatry* **60**, 1219-1229. doi: [10.1111/jcpp.13082](https://doi.org/10.1111/jcpp.13082)

**Lataster, J., van Os, J., de Haan, L., Thewissen, V., Bak, M., Lataster, T., … & Myin-Germeys, I.** (2011). Emotional experience and estimates of D2 receptor occupancy in psychotic patients treated with haloperidol, risperidone, or olanzapine: an experience sampling study. *Journal of Clinical Psychiatry* **72**, 1397-404. doi: [10.4088/JCP.09m05466yel](https://doi.org/10.4088/jcp.09m05466yel)

**Lavender, J. M., De Young, K. P., Anestis, M. D., Wonderlich, S. A., Crosby, R. D., Engel, S. G., …. & Le Grange, D.** (2013a). Associations between retrospective versus ecological momentary assessment measures of emotion and eating disorder symptoms in anorexia nervosa. *Journal of Psychiatric Research* **47**, 1514-20. doi: [10.1016/j.jpsychires.2013.06.021](https://doi.org/10.1016%2Fj.jpsychires.2013.06.021)

**Lavender, J. M., De Young, K. P., Wonderlich, S. A., Crosby, R. D., Engel, S. G., Mitchell, J. E., … & Le Grange, D.** (2013b). Daily patterns of anxiety in anorexia nervosa: associations with eating disorder behaviors in the natural environment. *Journal of Abnormal Psychology* **122**, 672-83. doi: [10.1037/a0031823](https://doi.org/10.1037/a0031823)

**Lavender, J. M., Utzinger, L. M., Crosby, R. D., Goldschmidt, A. B., Ellison, J., Wonderlich, S. A., … & Le Grange, D.** (2016). A naturalistic examination of the temporal patterns of affect and eating disorder behaviors in anorexia nervosa. *International Journal of Eating Disorders* **49**, 77-83. doi: [10.1002/eat.22447](https://psycnet.apa.org/doi/10.1002/eat.22447)

**Lavender, J. M., Wonderlich, S. A., Crosby, R. D., Engel, S. G., Mitchell, J. E., Crow, S., … & Le Grange, D.** (2013c). A naturalistic examination of body checking and dietary restriction in women with anorexia nervosa. *Behavior Research and Therapy* **51**, 507-11. doi: [10.1016/j.brat.2013.05.004](https://doi.org/10.1016/j.brat.2013.05.004)

**Lavender, J. M., Wonderlich, S. A., Crosby, R. D., Engel, S. G., Mitchell, J. E., Crow, S. J., … & Le Grange, D.** (2013d). Personality-based subtypes of anorexia nervosa: examining validity and utility using baseline clinical variables and ecological momentary assessment. *Behavior Research and Therapy* **51**, 512-7. doi: [10.1016/j.brat.2013.05.007](https://doi.org/10.1016/j.brat.2013.05.007)

**Le Grange, D., Crosby, R. D., Engel, S. G., Cao, L., Ndungu, A., Crow, S. J., … & Wonderlich, S. A.** (2013). DSM-IV-defined anorexia nervosa versus subthreshold anorexia nervosa (EDNOS-AN). *European Eating Disorders Review* **21**, 1-7. doi: [10.1002/erv.2192](https://psycnet.apa.org/doi/10.1002/erv.2192)

**Leahey, T. M., Crowther, J. H. & Ciesla, J. A.** (2011). An ecological momentary assessment of the effects of weight and shape social comparisons on women with eating pathology, high body dissatisfaction, and low body dissatisfaction. *Behavior Therapy* **42**, 197-210. doi: [10.1016/j.beth.2010.07.003](https://doi.org/10.1016/j.beth.2010.07.003)

**Leendertse, P., Myin-Germeys, I., Lataster, T., Simons, C. J. P., Oorschot, M., Lardinois, M., … & G.R.O.U.P. Investigators** (2018). Subjective quality of life in psychosis: Evidence for an association with real world functioning? *Psychiatry Research* **261**, 116-123. doi: [10.1016/j.psychres.2017.11.074](https://doi.org/10.1016/j.psychres.2017.11.074)

**Leraas, B. C., Smith, K. E., Utzinger, L. M., Cao, L., Engel, S. G., Crosby, R. D., … & Wonderlich, S. A.** (2018). Affect-based profiles of bulimia nervosa: The utility and validity of indicators assessed in the natural environment. *Psychiatry Research* **259**, 210-215. doi: [10.1016/j.psychres.2017.09.080](https://psycnet.apa.org/doi/10.1016/j.psychres.2017.09.080)

**Lovato, N., Lack, L., Wright, H. & Kennaway, D. J.** (2014). Evaluation of a brief treatment program of cognitive behavior therapy for insomnia in older adults. *Sleep* **37**, 117-26. doi: [10.5665/sleep.3320](https://doi.org/10.5665%2Fsleep.3320)

**Mackesy-Amiti, M. E. & Donenberg, G.** (2020). Negative affect and emotion dysregulation among people who inject drugs: An ecological momentary assessment study. *Psychology of Addictive Behaviors* **34**, 650-659. doi: [10.1037/adb0000577](https://psycnet.apa.org/doi/10.1037/adb0000577)

**Mason, T. B., Lavender, J. M., Wonderlich, S. A., Crosby, R. D., Engel, S. G., Mitchell, J. E.,… & Peterson, C. B.** (2018). Examining a momentary mediation model of appearance-related stress, anxiety, and eating disorder behaviors in adult anorexia nervosa. *Eating and Weight Disorders* **23**, 637-644. doi: [10.1007/s40519-017-0404-y](https://psycnet.apa.org/doi/10.1007/s40519-017-0404-y)

**Mason, T. B., Lavender, J. M., Wonderlich, S. A., Steiger, H., Cao, L., Engel, S. G., … & Crosby, R. D.** (2017). Comfortably Numb: The Role of Momentary Dissociation in the Experience of Negative Affect Around Binge Eating. *Journal of Nervous and Mental Disorders* **205**, 335-339. doi: [10.1097/NMD.0000000000000658](https://psycnet.apa.org/doi/10.1097/NMD.0000000000000658)

**McCrae, C. S., Williams, J., Roditi, D., Anderson, R., Mundt, J. M., Miller, M. B., … & Robinson, M. E.** (2019). Cognitive behavioral treatments for insomnia and pain in adults with comorbid chronic insomnia and fibromyalgia: clinical outcomes from the SPIN randomized controlled trial. *Sleep* **42**, 01. doi: [10.1093/sleep/zsy234](https://doi.org/10.1093/sleep/zsy234)

**McKenna, B. S., Drummond, S. P. & Eyler, L. T.** (2014). Associations between circadian activity rhythms and functional brain abnormalities among euthymic bipolar patients: a preliminary study. *Journal of Affective Disorders* **164**, 101-6. doi: [10.1016/j.jad.2014.04.034](https://doi.org/10.1016%2Fj.jad.2014.04.034)

**McKone, K. M. P., Kennedy, T. M., Piasecki, T. M., Molina, B. S. G. & Pedersen, S. L.** (2019). In-the-Moment Drinking Characteristics: An Examination Across Attention-Deficit/Hyperactivity Disorder History and Race. *Alcohol: Clinical and Experimental Research* **43**, 1273-1283. doi: [10.1111/acer.14050](https://psycnet.apa.org/doi/10.1111/acer.14050)

**McMakin, D. L., Ricketts, E. J., Forbes, E. E., Silk, J. S., Ladouceur, C. D., Siegle, G. J., …& Dahl, R. E.** (2019). Anxiety Treatment and Targeted Sleep Enhancement to Address Sleep Disturbance in Pre/Early Adolescents with Anxiety. *Journal of Clinical Child and Adolescent Psychology* **48**, S284-S297. doi: [10.1080/15374416.2018.1463534](https://doi.org/10.1080/15374416.2018.1463534)

**Merikangas, K. R., Swendsen, J., Hickie, I. B., Cui, L., Shou, H., Merikangas, A. K., … & Zipunnikov, V.** (2019). Real-time Mobile Monitoring of the Dynamic Associations Among Motor Activity, Energy, Mood, and Sleep in Adults With Bipolar Disorder. *JAMA Psychiatry* **76**, 190-198. doi: 10.1001/jamapsychiatry.2018.3546

**Mokkink, L. B., Terwee, C. B., Patrick, D. L., Alonso, J., Stratford, P. W., Knol, D. L., … & de Vet, H. C.** (2010). The COSMIN study reached international consensus on taxonomy, terminology, and definitions of measurement properties for health-related patient-reported outcomes. *Journal of Clinical Epidemioloy* **63**, 737-45. doi: [10.1016/j.jclinepi.2010.02.006](https://doi.org/10.1016/j.jclinepi.2010.02.006)

**Morgan, J. K., Lee, G. E., Wright, A. G. C., Gilchrist, D. E., Forbes, E. E., McMakin, D. L., … & Silk, J. S.** (2017). Altered Positive Affect in Clinically Anxious Youth: the Role of Social Context and Anxiety Subtype. *Journal of Abnormal Child Psychology* **45**, 1461-1472. doi: 10.1007/s10802-016-0256-3

**Muehlenkamp, J. J., Engel, S. G., Wadeson, A., Crosby, R. D., Wonderlich, S. A., Simonich, H. & Mitchell, J. E.** (2009). Emotional states preceding and following acts of non-suicidal self-injury in bulimia nervosa patients. *Behavior Research and Therapy* **47**, 83-7. doi: [10.1016/j.brat.2008.10.011](https://doi.org/10.1016/j.brat.2008.10.011)

**Oorschot, M., Lataster, T., Thewissen, V., Lardinois, M., van Os, J., Delespaul, P. A. & Myin-Germeys, I.** (2012). Symptomatic remission in psychosis and real-life functioning. *British Journal of Psychiatry* **201**, 215-20. doi:10.1192/bjp.bp.111.104414

**Oorschot, M., Lataster, T., Thewissen, V., Lardinois, M., Wichers, M., van Os, J., ... & Myin-Germeys, I.** (2013). Emotional experience in negative symptoms of schizophrenia--no evidence for a generalized hedonic deficit. *Schizophrenia Bulletin* **39**, 217-25. doi: [10.1093/schbul/sbr137](https://doi.org/10.1093/schbul/sbr137)

**Owens, J., Sangal, R. B., Sutton, V. K., Bakken, R., Allen, A. J. & Kelsey, D.** (2009). Subjective and objective measures of sleep in children with attention-deficit/hyperactivity disorder. *Sleep Medicine* **10**, 446-56.doi: [10.1016/j.sleep.2008.03.013](https://doi.org/10.1016/j.sleep.2008.03.013)

**Pagani, L., St Clair, P. A., Teshiba, T. M., Service, S. K., Fears, S. C., Araya, C., … & Freimer, N. B.** (2016). Genetic contributions to circadian activity rhythm and sleep pattern phenotypes in pedigrees segregating for severe bipolar disorder. *Proceedings of the National Academy of Science U S A* **113**, E754-61. doi: [10.1073/pnas.1513525113](https://doi.org/10.1073/pnas.1513525113)

**Palmer, C. A., Clementi, M. A., Meers, J. M. & Alfano, C. A.** (2018). Co-Sleeping among School-Aged Anxious and Non-Anxious Children: Associations with Sleep Variability and Timing. *Journal of Abnormal Child Psychology* **46**, 1321-1332. doi: 10.1007/s10802-017-0387-1

**Pearson, C. M., Lavender, J. M., Cao, L., Wonderlich, S. A., Crosby, R. D., Engel, S. G., … & Crow, S. J.** (2017). Associations of borderline personality disorder traits with stressful events and emotional reactivity in women with bulimia nervosa. *Journal of Abnormal Psychology* **126**, 531-539. doi: [10.1037/abn0000225](https://psycnet.apa.org/doi/10.1037/abn0000225)

**Pearson, C. M., Pisetsky, E. M., Goldschmidt, A. B., Lavender, J. M., Wonderlich, S. A., Crosby, R. D., … & Peterson, C. B.** (2016). Personality psychopathology differentiates risky behaviors among women with bulimia nervosa. *International Journal of Eating Disorders* **49**, 681-8. doi: [10.1002/eat.22570](https://doi.org/10.1002/eat.22570)

**Peerbooms, O., Rutten, B. P., Collip, D., Lardinois, M., Lataster, T., Thewissen, … & van Winkel, R.** (2012). Evidence that interactive effects of COMT and MTHFR moderate psychotic response to environmental stress. *Acta Psychiatrica Scandinavica* **125**, 247-56. doi: [10.1111/j.1600-0447.2011.01806.x](https://doi.org/10.1111/j.1600-0447.2011.01806.x)

**Perez Arribas, I., Goodwin, G. M., Geddes, J. R., Lyons, T. & Saunders, K. E. A.** (2018). A signature-based machine learning model for distinguishing bipolar disorder and borderline personality disorder. *Translational Psychiatry* **8**, 274. doi: 10.1038/s41398-018-0334-0

**Piette, J. D., Richardson, C., Himle, J., Duffy, S., Torres, T., Vogel, M., … & Valenstein, M.** (2011a). A randomized trial of telephonic counseling plus walking for depressed diabetes patients. *Medical Care* **49**, 641-8. doi: [10.1097/MLR.0b013e318215d0c9](https://doi.org/10.1097%2FMLR.0b013e318215d0c9)

**Piette, J. D., Valenstein, M., Himle, J., Duffy, S., Torres, T., Vogel, M. & Richardson, C.** (2011b). Clinical complexity and the effectiveness of an intervention for depressed diabetes patients. *Chronic Illness* **7**, 267-78. doi: [10.1177/174239531140925](https://doi.org/10.1177/1742395311409259)

**Pillai, V., Steenburg, L. A., Ciesla, J. A., Roth, T. & Drake, C. L.** (2014). A seven day actigraphy-based study of rumination and sleep disturbance among young adults with depressive symptoms. *Journal of Psychosomatic Research* **77**, 70-5. doi: [10.1016/j.jpsychores.2014.05.004](https://doi.org/10.1016/j.jpsychores.2014.05.004)

**Pinkham, A. E., Ackerman, R. A., Depp, C. A., Harvey, P. D. & Moore, R. C.** (2020). A Longitudinal Investigation of the Effects of the COVID-19 Pandemic on the Mental Health of Individuals with Pre-existing Severe Mental Illnesses. *Psychiatry Research* **294**, 113493. doi: [10.1016/j.psychres.2020.113493](https://doi.org/10.1016/j.psychres.2020.113493)

**Pisetsky, E. M., Crosby, R. D., Cao, L., Fitzsimmons-Craft, E. E., Mitchell, J. E., Engel, S. G., … & Peterson, C. B.** (2016). An examination of affect prior to and following episodes of getting drunk in women with bulimia nervosa. *Psychiatry Research* **240**, 202-208. doi: [10.1016/j.psychres.2016.04.044](https://doi.org/10.1016/j.psychres.2016.04.044)

**Pratap, A., Atkins, D. C., Renn, B. N., Tanana, M. J., Mooney, S. D., Anguera, J. A. & Arean, P. A.** (2019). The accuracy of passive phone sensors in predicting daily mood. *Depression and Anxiety* **36**, 72-81. doi: [10.1002/da.22822](https://doi.org/10.1002/da.22822)

**Primack, B. A., Silk, J. S., DeLozier, C. R., Shadel, W. G., Dillman Carpentier, F. R., Dahl, R. E. & Switzer, G. E.** (2011). Using ecological momentary assessment to determine media use by individuals with and without major depressive disorder. *Archives of Pediatric and Adolescent Medicine* **165**, 360-5. doi: 10.1001/archpediatrics.2011.27

**Prunas, C., Krane-Gartiser, K., Nevoret, C., Benard, V., Benizri, C., Brochard, H., … & Etain, B.** (2019). Does childhood experience of attention-deficit hyperactivity disorder symptoms increase sleep/wake cycle disturbances as measured with actigraphy in adult patients with bipolar disorder? *Chronobiology International* **36**, 1124-1130. doi: [10.1080/07420528.2019.1619182](https://doi.org/10.1080/07420528.2019.1619182)

**Ranum, B. M., Wichstrom, L., Pallesen, S., Falch-Madsen, J., Halse, M. & Steinsbekk, S.** (2019). Association Between Objectively Measured Sleep Duration and Symptoms of Psychiatric Disorders in Middle Childhood. *JAMA Network Open* **2**, e1918281. doi: 10.1001/jamanetworkopen.2019.18281

**Reece, A. G., Reagan, A. J., Lix, K. L. M., Dodds, P. S., Danforth, C. M. & Langer, E. J.** (2017). Forecasting the onset and course of mental illness with Twitter data. *Scientific Reports* **7**, 13006. doi: 10.1038/s41598-017-12961-9

**Reininghaus, U., Gayer-Anderson, C., Valmaggia, L., Kempton, M. J., Calem, M., Onyejiaka, A.,… & Morgan, C.** (2016a). Psychological processes underlying the association between childhood trauma and psychosis in daily life: an experience sampling study. *Psychological Medicine* **46**, 2799-813. doi: 10.1017/S003329171600146X

**Reininghaus, U., Kempton, M. J., Valmaggia, L., Craig, T. K., Garety, P., Onyejiaka, A.,… & Morgan, C.** (2016b). Stress Sensitivity, Aberrant Salience, and Threat Anticipation in Early Psychosis: An Experience Sampling Study. *Schizophrenia Bulletin* **42**, 712-22. doi: 10.1017/S003329171600146X

**Rintala, A., Wampers, M., Myin-Germeys, I. & Viechtbauer, W.** (2019). Response compliance and predictors thereof in studies using the experience sampling method. *Psychological Assessment* **31**, 226-235.doi: [10.1037/pas0000662](https://psycnet.apa.org/doi/10.1037/pas0000662)

**Robillard, R., Hermens, D. F., Naismith, S. L., White, D., Rogers, N. L., Ip, T. K., ... & Hickie, I. B.** (2015). Ambulatory sleep-wake patterns and variability in young people with emerging mental disorders. *Journal of Psychiatry and Neuroscience* **40**, 28-37. doi: 10.1503/jpn.130247

**Robillard, R., Naismith, S. L., Smith, K. L., Rogers, N. L., White, D., Terpening, Z., ... & Hickie, I. B.** (2014). Sleep-wake cycle in young and older persons with a lifetime history of mood disorders. *PLoS One* **9**, e87763. doi: [10.1371/journal.pone.0087763](https://doi.org/10.1371/journal.pone.0087763)

**Robillard, R., Oxley, C., Hermens, D. F., White, D., Wallis, R., Naismith, S. L., … & Hickie, I. B.** (2016). The relative contributions of psychiatric symptoms and psychotropic medications on the sleep-wake profile of young persons with anxiety, depression and bipolar disorders. *Psychiatry Research* **243**, 403-6. doi: [10.1016/j.psychres.2016.06.025](https://doi.org/10.1016/j.psychres.2016.06.025)

**Rusby, J. C., Westling, E., Crowley, R., Mills, K. L. & Light, J. M.** (2019). Associations between marijuana use and anxious mood lability during adolescence. *Addiction and Behavior* **92**, 89-94. doi: [10.1016/j.addbeh.2018.12.029](https://doi.org/10.1016/j.addbeh.2018.12.029)

**Ruscio, A. M., Gentes, E. L., Jones, J. D., Hallion, L. S., Coleman, E. S. & Swendsen, J.** (2015). Rumination predicts heightened responding to stressful life events in major depressive disorder and generalized anxiety disorder. *Journal of Abnormal Psychology* **124**, 17-26. doi: [10.1037/abn0000025](https://psycnet.apa.org/doi/10.1037/abn0000025)

**Sauchelli, S., Arcelus, J., Sanchez, I., Riesco, N., Jimenez-Murcia, S., Granero, R., ... & Fernandez-Aranda, F.** (2015). Physical activity in anorexia nervosa: How relevant is it to therapy response? *European Psychiatry* **30**, 924-31. doi: 10.1016/j.eurpsy.2015.09.008

**Schaefer, L. M., Smith, K. E., Anderson, L. M., Cao, L., Crosby, R. D., Engel, S. G., … & Wonderlich, S. A.** (2020). The role of affect in the maintenance of binge-eating disorder: Evidence from an ecological momentary assessment study. *Journal of Abnormal Psychology* **129**, 387-396. doi: [10.1037/abn0000517](https://psycnet.apa.org/doi/10.1037/abn0000517)

**Schlam, T. R., Baker, T. B., Smith, S. S., Cook, J. W. & Piper, M. E.** (2020). Anxiety Sensitivity and Distress Tolerance in Smokers: Relations With Tobacco Dependence, Withdrawal, and Quitting Successdagger. *Nicotine and Tobacco Research* **22**, 58-65. doi: [10.1093/ntr/ntz070](https://doi.org/10.1093/ntr/ntz070)

**Schreiber-Gregory, D. N., Lavender, J. M., Engel, S. G., Wonderlich, S. A., Crosby, R. D., Peterson, C. B., ... & Mitchell, J. E.** (2013). Examining duration of binge eating episodes in binge eating disorder. *International Journal of Eating Disorders* **46**, 810-4. doi: [**10.1002/eat.22164**](https://doi.org/10.1002/eat.22164)

**Scott, L. N., Stepp, S. D., Hallquist, M. N., Whalen, D. J., Wright, A. G. C. & Pilkonis, P. A.** (2015). Daily shame and hostile irritability in adolescent girls with borderline personality disorder symptoms. *Personal Disorders* **6**, 53-63. doi: [10.1037/per0000107](https://psycnet.apa.org/doi/10.1037/per0000107)

**Scott, L. N., Wright, A. G. C., Beeney, J. E., Lazarus, S. A., Pilkonis, P. A. & Stepp, S. D.** (2017). Borderline personality disorder symptoms and aggression: A within-person process model. *Journal of Abnormal Psychology* **126**, 429-440. doi: [10.1037/abn0000272](https://psycnet.apa.org/doi/10.1037/abn0000272)

**Selby, E. A., Doyle, P., Crosby, R. D., Wonderlich, S. A., Engel, S. G., Mitchell, J. D. & Le Grange, D.** (2012). Momentary emotion surrounding bulimic behaviors in women with bulimia nervosa and borderline personality disorder. *Journal of Psychiatric Research* **46**, 1492-500. doi: [10.1016/j.jpsychires.2012.08.014](https://doi.org/10.1016/j.jpsychires.2012.08.014)

**Serre, F., Fatseas, M., Denis, C., Swendsen, J. & Auriacombe, M.** (2018). Predictors of craving and substance use among patients with alcohol, tobacco, cannabis or opiate addictions: Commonalities and specificities across substances. *Addiction and Behavior* **83**, 123-129. doi: [10.1016/j.addbeh.2018.01.041](https://doi.org/10.1016/j.addbeh.2018.01.041)

**Shou, H., Cui, L., Hickie, I., Lameira, D., Lamers, F., Zhang, J., ... & Merikangas, K. R.** (2017). Dysregulation of objectively assessed 24-hour motor activity patterns as a potential marker for bipolar I disorder: results of a community-based family study. *Translational Psychiatry* **7**, e1211. doi: 10.1038/tp.2017.136

**Silk, J. S., Tan, P. Z., Ladouceur, C. D., Meller, S., Siegle, G. J., McMakin, D. L., ... & Ryan, N. D.** (2018). A Randomized Clinical Trial Comparing Individual Cognitive Behavioral Therapy and Child-Centered Therapy for Child Anxiety Disorders. *Journal of Clinical Child and Adolescent Psychology* **47**, 542-554. doi: [10.1080/15374416.2016.1138408](https://doi.org/10.1080/15374416.2016.1138408)

**Simons, C. J., Hartmann, J. A., Kramer, I., Menne-Lothmann, C., Hohn, P., van Bemmel, A. L., ... & Wichers, M.** (2015). Effects of momentary self-monitoring on empowerment in a randomized controlled trial in patients with depression. *European Psychiatry* **30**, 900-6. doi: 10.1016/j.eurpsy.2015.09.004

**Slyepchenko, A., Allega, O. R., Leng, X., Minuzzi, L., Eltayebani, M. M., Skelly, M., ... & Frey, B. N.** (2019). Association of functioning and quality of life with objective and subjective measures of sleep and biological rhythms in major depressive and bipolar disorder. *Australian and New Zeeland Journal of Psychiatry* **53**, 683-696. doi: [10.1177/00048674198292](https://doi.org/10.1177/0004867419829228)

**Smagula, S. F., DuPont, C. M., Miller, M. A., Krafty, R. T., Hasler, B. P., Franzen, P. L. & Roecklein, K. A.** (2018). Rest-activity rhythms characteristics and seasonal changes in seasonal affective disorder. *Chronobiology International* **35**, 1553-1559. doi: [10.1080/07420528.2018.1496094](https://doi.org/10.1080/07420528.2018.1496094)

**Smyth, J. M., Wonderlich, S. A., Sliwinski, M. J., Crosby, R. D., Engel, S. G., Mitchell, J. E. & Calogero, R. M.** (2009). Ecological momentary assessment of affect, stress, and binge-purge behaviors: day of week and time of day effects in the natural environment. *International Journal of Eating Disorders* **42**, 429-36. doi: [**10.1002/eat.20623**](https://doi.org/10.1002/eat.20623)

**Solhan, M. B., Trull, T. J., Jahng, S. & Wood, P. K.** (2009). Clinical assessment of affective instability: comparing EMA indices, questionnaire reports, and retrospective recall. *Psychological Assessment* **21**, 425-36. doi: [10.1037/a0016869](https://psycnet.apa.org/doi/10.1037/a0016869)

**Soreca, I., Wallace, M. L., Hall, M. H., Hasler, B. P., Frank, E. & Kupfer, D. J.** (2016). The association between meal timing and frequency with cardiometabolic profile in patients with bipolar disorder. *Acta Psychiatrica Scandinavica* **133**, 453-8. doi: [**10.1111/acps.12578**](https://doi.org/10.1111/acps.12578)

**Sperry, S. H., Walsh, M. A. & Kwapil, T. R.** (2020). Emotion dynamics concurrently and prospectively predict mood psychopathology. *Journal of Affective Disorders* **261**, 67-75. doi: [10.1016/j.jad.2019.09.076](https://doi.org/10.1016/j.jad.2019.09.076)

**Stubbs, B., Ku, P. W., Chung, M. S. & Chen, L. J.** (2017). Relationship Between Objectively Measured Sedentary Behavior and Cognitive Performance in Patients With Schizophrenia Vs Controls. *Schizophrenia Bulletin* **43**, 566-574. doi: [10.1093/schbul/sbw126](https://doi.org/10.1093/schbul/sbw126)

**Tasca, G. A., Illing, V., Balfour, L., Krysanski, V., Demidenko, N., Nowakowski, J. & Bissada, H.** (2009). Psychometric properties of self-monitoring of eating disorder urges among treatment seeking women: ecological momentary assessment using a daily diary method. *Eating Behavior* **10**, 59-61. doi: [10.1016/j.eatbeh.2008.10.004](https://doi.org/10.1016/j.eatbeh.2008.10.004)

**Thewissen, V., Bentall, R. P., Lecomte, T., van Os, J. & Myin-Germeys, I.** (2008). Fluctuations in self-esteem and paranoia in the context of daily life. *Journal of Abnormal Psychology* **117**, 143-53. doi: [10.1037/0021-843X.117.1.143](https://psycnet.apa.org/doi/10.1037/0021-843X.117.1.143)

**Thewissen, V., Bentall, R. P., Oorschot, M., J, A. C., van Lierop, T., van Os, J. & Myin-Germeys, I.** (2011). Emotions, self-esteem, and paranoid episodes: an experience sampling study. *British Journal of Clinical Psychology* **50**, 178-95. doi: [**10.1348/014466510X508677**](https://doi.org/10.1348/014466510X508677)

**Titone, M. K., McArthur, B. A., Ng, T. H., Burke, T. A., McLaughlin, L. E., MacMullen, L. E., ... & Alloy, L. B.** (2020). Sex and race influence objective and self-report sleep and circadian measures in emerging adults independently of risk for bipolar spectrum disorder. *Scientific Reports* **10**, 13731. doi: 10.1038/s41598-020-70750-3

**Troxel, W. M., Buysse, D. J., Monk, T. H., Begley, A. & Hall, M.** (2010). Does social support differentially affect sleep in older adults with versus without insomnia? *Journal of Psychosomatic Research* **69**, 459-66. doi: [10.1016/j.jpsychores.2010.04.003](https://doi.org/10.1016/j.jpsychores.2010.04.003)

**Tsanas, A., Saunders, K. E., Bilderbeck, A. C., Palmius, N., Osipov, M., Clifford, G. D., ... & De Vos, M.** (2016). Daily longitudinal self-monitoring of mood variability in bipolar disorder and borderline personality disorder. *Journal of Affective Disorders* **205**, 225-233. doi: [10.1016/j.jad.2016.06.065](https://doi.org/10.1016/j.jad.2016.06.065)

**Udachina, A., Bentall, R. P., Varese, F. & Rowse, G.** (2017). Stress sensitivity in paranoia: poor-me paranoia protects against the unpleasant effects of social stress. *Psychological Medicine* **47**, 2834-2843. doi: 10.1017/S0033291717001362

**Verkooijen, S., Stevelink, R., Abramovic, L., Vinkers, C. H., Ophoff, R. A., Kahn, R. S., ... & van Haren, N. E.** (2017). The association of sleep and physical activity with integrity of white matter microstructure in bipolar disorder patients and healthy controls. *Psychiatry Research: Neuroimaging* **262**, 71-80. doi: [10.1016/j.pscychresns.2017.01.013](https://doi.org/10.1016/j.pscychresns.2017.01.013)

**Von Korff, M., Vitiello, M. V., McCurry, S. M., Balderson, B. H., Moore, A. L., Baker, L. D., ... & Rybarczyk, B. D.** (2012). Group interventions for co-morbid insomnia and osteoarthritis pain in primary care: the lifestyles cluster randomized trial design. *Contemporary Clinical Trials* **33**, 759-68. doi: [10.1016/j.cct.2012.03.010](https://doi.org/10.1016/j.cct.2012.03.010)

**Wallace, M. L., McMakin, D. L., Tan, P. Z., Rosen, D., Forbes, E. E., Ladouceur, C. D., ... & Silk, J. S.** (2017). The role of day-to-day emotions, sleep, and social interactions in pediatric anxiety treatment. *Behavior Research and Therapy* **90**, 87-95. doi: [10.1016/j.brat.2016.12.012](https://doi.org/10.1016/j.brat.2016.12.012)

**Wallen, G. R., Park, J., Krumlauf, M. & Brooks, A. T.** (2019). Identification of Distinct Latent Classes Related to Sleep, PTSD, Depression, and Anxiety in Individuals Diagnosed With Severe Alcohol Use Disorder. *Behavior Sleep Medicine* **17**, 514-523. doi: [10.1080/15402002.2018.1425867](https://doi.org/10.1080/15402002.2018.1425867)

**Walther, S., Ramseyer, F., Horn, H., Strik, W. & Tschacher, W.** (2014). Less structured movement patterns predict severity of positive syndrome, excitement, and disorganization. *Schizoprenia Bulletin* **40**, 585-91. doi: [10.1093/schbul/sbt038](https://doi.org/10.1093/schbul/sbt038)

**Wichers, M., Myin-Germeys, I., Jacobs, N., Peeters, F., Kenis, G., Derom, C., ... & Van Os, J.** (2007). Genetic risk of depression and stress-induced negative affect in daily life. *British Journal of Psychiatry* **191**, 218-23. doi: 10.1192/bjp.bp.106.032201

**Wichniak, A., Skowerska, A., Chojnacka-Wojtowicz, J., Taflinski, T., Wierzbicka, A., Jernajczyk, W. & Jarema, M.** (2011). Actigraphic monitoring of activity and rest in schizophrenic patients treated with olanzapine or risperidone. *Journal of Psychiatric Research* **45**, 1381-6. doi: [10.1016/j.jpsychires.2011.05.009](https://doi.org/10.1016/j.jpsychires.2011.05.009)

**Wigman, J. T., van Os, J., Borsboom, D., Wardenaar, K. J., Epskamp, S., Klippel, A., ... & Wichers, M.** (2015). Exploring the underlying structure of mental disorders: cross-diagnostic differences and similarities from a network perspective using both a top-down and a bottom-up approach. *Psychological Medicine* **45**, 2375-87. doi: 10.1017/S0033291715000331

**Williams-Kerver, G. A. & Crowther, J. H.** (2020). Emotion differentiation and disordered eating behaviors: The role of appearance schemas. *Eating Behavior* **37**, 101369. doi: [10.1016/j.eatbeh.2020.101369](https://doi.org/10.1016/j.eatbeh.2020.101369)

**Wonderlich, J. A., Lavender, J. M., Wonderlich, S. A., Peterson, C. B., Crow, S. J., Engel, S. G., ... & Crosby, R. D.** (2015). Examining convergence of retrospective and ecological momentary assessment measures of negative affect and eating disorder behaviors. *International Journal of Eating Disorders* **48**, 305-11. doi: [10.1002/eat.22352](https://doi.org/10.1002/eat.22352)

**Wonderlich, S. A., Rosenfeldt, S., Crosby, R. D., Mitchell, J. E., Engel, S. G., Smyth, J. & Miltenberger, R.** (2007). The effects of childhood trauma on daily mood lability and comorbid psychopathology in bulimia nervosa. *Journal of Traumatic Stress* **20**, 77-87. doi: [10.1002/jts.20184](https://doi.org/10.1002/jts.20184)

**Yaroslavsky, I., Napolitano, S. C. & France, C. M.** (2019). Ruminative responses to interpersonal precipitants mediate borderline personality disorder features' effects on distress reactivity and recovery in daily life. *Journal of Clinical Psychology* **75**, 2188-2209. doi: [10.1002/jclp.22839](https://doi.org/10.1002/jclp.22839)

**Yeung, W. F., Chung, K. F., Tso, K. C., Zhang, S. P., Zhang, Z. J. & Ho, L. M.** (2011). Electroacupuncture for residual insomnia associated with major depressive disorder: a randomized controlled trial. *Sleep* **34**, 807-15. doi: [10.5665/SLEEP.1056](https://doi.org/10.5665/SLEEP.1056)

**Yin, X., Gou, M., Xu, J., Dong, B., Yin, P., Masquelin, F., ... & Xu, S.** (2017). Efficacy and safety of acupuncture treatment on primary insomnia: a randomized controlled trial. *Sleep Medicine* **37**, 193-200. doi: [10.1016/j.sleep.2017.02.012](https://doi.org/10.1016/j.sleep.2017.02.012)

**Zunker, C., Peterson, C. B., Crosby, R. D., Cao, L., Engel, S. G., Mitchell, J. E. & Wonderlich, S. A.** (2011). Ecological momentary assessment of bulimia nervosa: does dietary restriction predict binge eating? *Behavior Research and Therapy* **49**, 714-7. doi: [10.1016/j.brat.2011.06.006](https://doi.org/10.1016/j.brat.2011.06.006)
